# The intracellular domain of TLR2 is capable of high‐affinity Zn binding: possible outcomes for the receptor activation

**DOI:** 10.1002/1873-3468.70026

**Published:** 2025-03-03

**Authors:** Vladislav A. Lushpa, Cong Lin, Irina A. Talyzina, Marina V. Goncharuk, Eduard V. Bocharov, Alexander S. Arseniev, Xiaohui Wang, Sergey A. Goncharuk, Konstantin S. Mineev

**Affiliations:** ^1^ Shemyakin‐Ovchinnikov Institute of Bioorganic Chemistry RAS Moscow Russia; ^2^ Moscow Center for Advanced Studies Moscow Russia; ^3^ Laboratory of Chemical Biology, Changchun Institute of Applied Chemistry Chinese Academy of Sciences Changchun China; ^4^ Department of Applied Chemistry and Engineering University of Science and Technology of China Hefei China; ^5^ Present address: Department of Biochemistry and Molecular Biophysics Columbia University New York NY USA; ^6^ Present address: Institute of Organic Chemistry and Chemical Biology Goethe University Frankfurt Frankfurt am Main Germany

**Keywords:** NMR, oligomerization, activation, TIR domain, TLR2, zinc, Toll‐like receptor, zinc binding

## Abstract

Toll‐like receptors (TLRs) are important players in the innate immune system. Binding of pathogen‐related molecules to the extracellular domains of TLRs initiates signalosome assembly, a key event in signal transduction. Despite extensive research on individual receptor domains, the mechanism of signalosome assembly remains unclear. Recent evidence suggests that the intracellular TIR domain of TLR1 binds zinc ions, with cysteines playing a pivotal role in binding and receptor activation. This study explores the zinc‐binding ability of the TLR2 TIR domain (TLR2_TIR_). We found that TLR2_TIR_ binds zinc with nanomolar affinity through its cysteine residues. Two of them, C673 and C713, are essential for receptor activation. These results suggest that zinc may be involved in the initiation of signalosome assembly.

## Graphical abstract

Toll‐like receptors (TLRs) are important in the innate immune system. This study explores the zinc‐binding ability of the TLR2 TIR domain (TLR2_TIR_). We found that TLR2_TIR_ binds zinc with nanomolar affinity through its cysteine residues. Two of them, C673 and C713, are vital for receptor activation, indicating that zinc may play a role in initiating signalosome assembly.

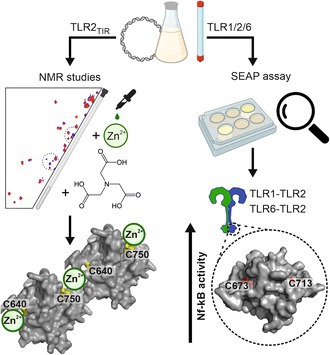

## Abbreviations

**AP‐1**, activator protein 1

**Cryo‐EM**, cryogenic electron microscopy

**DLS**, dynamic light scattering

**EDTA**, ethylenediaminetetraacetic acid

**EGTA**, triethylene glycol diamine tetraacetic acid

**FBS**, fetal bovine serum

**HSQC**, heteronuclear single quantum coherence spectroscopy

**IMAC**, immobilized metal affinity chromatography

**MOPS**, 3‐(*N*‐morpholino)propanesulfonic acid

**NEAA**, nonessential amino acid

**NF‐κB**, nuclear factor‐κB

**NMR**, nuclear magnetic resonance spectroscopy

**NTA**, nitrilotriacetic acid

**SEAP**, secreted embryonic alkaline phosphatase

**SEC**, size exclusion chromatography

**SYBR Green**, *N*′,*N*′‐dimethyl‐*N*‐[4‐[(E)‐(3‐methyl‐1,3‐benzothiazol‐2‐ylidene)methyl]‐1‐‐phenylquinolin‐1‐ium‐2‐‐yl]‐*N*‐propylpropane‐1,3‐diamine

**TCEP**, Tris(2‐carboxyethyl)phosphine

**TIR**, toll/interleukin‐1 receptor homology domain

**TLR**, toll‐like receptor

**WT**, wild‐type

The innate immune system plays a key role in protection against pathogenic microorganisms [1, 2]. One of the most important components of this system is the Toll‐like receptors (TLRs), which are responsible for recognizing pathogen‐associated molecular patterns from various pathogens and danger‐associated molecular patterns from the host [3, 4]. Many diseases, including various types of cancer, are associated with TLR pathologies [5, 6, 7, 8], and there are numerous TLR‐targeted drugs either already on the market or in clinical trials [9]. Overall, the molecular mechanism of activation is well understood: pathogen binding induces the formation of a dimeric TLR complex, which then interacts with a set of adapter proteins, leading to the assembly of a supramolecular complex called ‘Myddosome’. This complex is responsible for further signaling [10, 11, 12]. In particular, TLR2 is known to form heterodimeric complexes with TLR1, TLR6, or TLR10 upon binding to the fragments of bacterial, viral, or fungal cell walls [10, 13]. This heterodimer triggers a cascade of intracellular signaling events leading to the activation of the transcription factors NF‐κB and AP‐1 and initiates the expression of cytokines, interferons, etc. [10].

Recent studies of TLRs have provided significant insights into their structural organization. The PDB database now contains the structures of the extracellular domains for all TLR members [14, 15, 16, 17, 18, 19, 20], including their ligand‐bound states, as well as intracellular domains for TLR1/2/6/10 [21, 22, 23], and five transmembrane domains TLR2/3/4/5/9 [24, 25, 26]. Based on the structures of individual domains, models of full‐length receptors have been proposed [25, 27, 28], but their relevance remains unclear. Finally, attempts to determine the full‐length receptor structure for TLR5 [29] and TLR3/7 [30] were only partially successful. The structure of TLR5 was determined using the single‐particle image reconstruction technique of electron microscopy with a resolution of 26 Å. The structures of TLR3 and TLR7 were determined by cryo‐EM, but only the extracellular domains could be solved with a resolution of 3.1 Å, while the intracellular domains were completely absent in the cryo‐EM density map.

Thus, despite all the structural data, the atomistic details of TLR activation are still unclear. The main blind spot is the signal transduction from the TLRs to their adaptor proteins, since no structure of the TLR intracellular domain in complex with any of the adaptor proteins has been solved. Moreover, our recent research has shown that TLR1 activation involves the binding of Zn ions to its TIR domain [31], which further complicates the pathways of TLR activation. We found that cysteine residues in the TIR domain are essential for Zn binding and critical for receptor activation. These findings are consistent with numerous studies highlighting the important role of Zn in the TLR signaling pathway [32, 33, 34, 35, 36, 37]. Given the heterodimer nature of the TLR1/2 complex, we investigated the ability of the TIR domain of TLR2 (TLR2_TIR_) to bind Zn and the functional significance of this interaction.

## Materials and methods

### Gene assembling

The gene encoding the 636–784 fragment of human TLR2 (UniProtKB – O60603) was synthesized by Twist Bioscience (San Francisco, CA, USA) with partial codon optimization for expression in *Escherichia coli* and amplified using PCR. The mutations were introduced by PCR using chemically synthesized oligonucleotides (Table S1). The PCR products were cloned into the pGEMEX‐1 vector [38] using BamHI and HindIII restriction sites, resulting in the pG1‐TIR_TLR2_ vector. The gene of interest was verified by DNA sequencing (Evrogen, Moscow, Russia, Table S2).

### Protein production and purification

Protein production and purification protocols for TLR1_TIR_ and TLR2_TIR_ are described in detail in our previous works [39, 40, 41]. Briefly, the proteins were produced in BL21(DE3) pLysS *E. coli* strain, which was cultured at 28 °C for 24 h after the addition of 0.01 mm IPTG at OD = 0.6. M9 minimal salts medium containing ^15^NH_4_Cl was used to obtain the ^15^N‐labeled protein. After cell lysis, TLR1_TIR_ was purified by immobilized metal affinity chromatography (IMAC) using Ni^2+^ sepharose HP resin (GE Healthcare, Chicago, IL, USA), cation‐exchange chromatography using SP sepharose FF resin (GE Healthcare), and size exclusion chromatography (SEC) using a Tricorn 10/300 Superdex 75 increase column (GE Healthcare). TLR2_TIR_ (Table S3) was purified by IMAC, ion exchange chromatography on Q sepharose fast flow (GE Healthcare), and SEC. The purified protein samples were dialyzed against the NMR buffer and concentrated. For TLR2_TIR_ mutants (Table S3), the production and purification protocols developed for wild‐type (WT) TLR2_TIR_ were used.

### SEC and DLS analysis

500 μL of protein solution was injected into the calibrated GE 10/300 Superdex 200 increase column at 20 °C and 0.5 mL·min^−1^ to analyze the protein molecular weight. Simultaneously, a DLS experiment was performed on the same sample using a Wyatt DynaPro Titan spectrometer. For each sample, we performed at least five measurements, consisting of 20 acquisitions of 10 s each. These data were analyzed using the dynamics 6.7.7.9 software (Wyatt Technologies, Santa Barbara, CA, USA). The hydrodynamic radii were averaged between the measurements.

To study the effect of Zn ions, SEC fractions corresponding to the TLR2_TIR_ monomer were pooled, and ZnCl_2_ was added to the protein (~ 40 μm) at different Zn : protein ratios: 0 : 1, 0.5 : 1, 1 : 1, and 2 : 1. Samples were incubated at 25 °C for 6–7 h, and aggregates were pelleted down by centrifugation at 25 000 ***g*** at 20 °C for 1 h. The supernatant was injected onto the same SEC column at 20 °C and 0.5 mL·min^−1^.

### NMR spectroscopy

TLR2_TIR_ WT and its mutants were prepared in 450 μL of H_2_O with 5% D_2_O (Sigma‐Aldrich, St. Louis, MO, USA). Each sample contained 200 μm target protein, 30 mm MOPS, 64.4 mm KCl, 5.3 mm NaCl, 0.5 mm MgCl_2_, 1 mm TCEP, and 0.001% NaN_3_, pH 7.4. For each sample, a series of ^1^H and ^1^H,^15^N‐HSQC NMR spectra were recorded: for the sample itself, after adding an equimolar amount of NTA, and after titration with ZnCl_2_ from 0 to 200 μm. TLR2_TIR_ WT, C713A, C640/750A were additionally titrated with an equimolar mixture of NTA and ZnCl_2_ from 0 to 200 μm. NMR spectra were recorded on Bruker Avance 700 MHz or Bruker Avance III 600 MHz spectrometers equipped with triple resonance cryoprobes at 303 K. The recorded spectra were analyzed in the topspin v3.4 software (Bruker Biospin, Ettlingen, Germany). The concentration of the bound NTA was determined based on the ratio of the MOPS (2.22 ppm) and chelator (3.63 ppm) signals in the ^1^H NMR spectra. The concentration of free NTA was taken as the difference between the concentrations of total NTA and its zinc‐bound form. The concentration of the free form of TLR2_TIR_ was determined as the arithmetic mean of the protein concentrations calculated for the NH signals in the ^1^H,^15^N‐HSQC NMR spectrum using the formula:

$$\left[\mathrm{apo}\;\mathrm{TLR}2_{\mathrm{TIR}}\right]=\left[\text{control}\;\mathrm{TLR}2_{\mathrm{TIR}}\right]\times \frac{\text{signals}\;\mathrm{TLR}2_{\mathrm{TIR}}\;\text{intensity}}{\text{control signals}\;\mathrm{TLR}2_{\mathrm{TIR}}\;\text{intensity}},$$

where [$\text{control}\;\mathrm{TLR}2_{\mathrm{TIR}}$] is the concentration of TLR2_TIR_ measured spectrophotometrically, signals TLR2_TIR_ intensity – intensity of NH signal of TLR2_TIR_ in ^1^H,^15^N‐HSQC spectra and control signals TLR2_TIR_ intensity – intensities of NH signals recorded for the sample in the absence of Zn. To analyze the intensity changes, we selected six cross‐peaks (Fig. S1) according to two criteria: (a) the signal should be a distinct peak in the spectrum; (b) the peak should be detectable in all experiments (signal‐to‐noise ratio greater than 4 for all titration points). The concentrations of apo TLR2_TIR_ were measured for cross‐peaks in ^1^H,^15^N‐HSQC and then averaged. The error of each measurement includes the sum of the standard deviation and the correction for protein sedimentation over time. The correction was determined based on the dependence of signal intensity on time in the absence of zinc (Fig. S2).

To assess the interaction between TLR1_TIR_ and TLR2_TIR_, two experiments were performed. First, the equimolar mixture of TLR1_TIR_ and TLR2_TIR_ was titrated with zinc. 100 μm samples of TLR1_TIR_ and TLR2_TIR_ were used. The 100 μm solution of TLR2_TIR_ was added to the 100 μm solution of TLR1_TIR_, and the mixture was concentrated twofold. Then, 50 and 100 μm zinc were added to the equimolar mixture of proteins. Each step was monitored by NMR spectroscopy. A 100 μm sample of TLR1_TIR_ was used as a control. Second, the equimolar mixture of TLR1_TIR_ and Zn was titrated with TLR2_TIR_. A 400 μm stock solution of TLR2_TIR_ was added to a 100 μm sample of the mixture of TLR1_TIR_ and zinc until TLR1_TIR_ : TLR2_TIR_ ratios of 1 : 4, 1 : 2, 3 : 4, and 1 : 1 were reached. ^1^H, ^1^H,^15^N‐HSQC NMR spectra were recorded at each titration step.

For the EDTA competition assay, two 200 μm TLR2_TIR_ samples were prepared. For the first sample, ^1^H,^15^N‐HSQC NMR spectra were recorded without Zn and immediately after the addition of an equimolar amount of ZnCl_2_. After 1.5‐h incubation, 600 μm EDTA was added to the TLR2_TIR_ : Zn mixture, and the same spectrum was acquired. For the second sample, zinc was added to the protein, and the mixture was incubated for 24 h. 600 μm EDTA was then added to the sample, and the same spectrum was recorded immediately, after 48 h, and after 96 h.

### Data analysis

The dissociation constants of WT TLR2_TIR_ and its mutants with zinc were determined in the wolfram mathematica 5.0 software (Wolfram Research, Champaign, IL, USA)  based on experimental data. The dissociation constant was obtained by solving the system of equations:

$$\left[{\mathrm{Zn}}_{\mathrm{TLR}2}\right]+\left[{\mathrm{Zn}}_{\text{chelator}}\right]+\left[{\mathrm{Zn}}_{\text{free}}\right]=\left[{\mathrm{Zn}}_{\text{total}}\right]$$

$$\left[\mathrm{TLR}2_{\mathrm{Zn}}\right]+\left[\mathrm{TLR}2_{\text{free}}\right]=\left[\mathrm{TLR}2_{\text{total}}\right]$$

$$\left[{\mathrm{NTA}}_{\mathrm{Zn}}\right]+\left[{\mathrm{NTA}}_{\text{free}}\right]=\left[{\mathrm{NTA}}_{\text{total}}\right]$$

$$\left[{\mathrm{Zn}}_{\mathrm{TLR}2}\right]=\left[\mathrm{TLR}2_{\mathrm{Zn}}\right]$$

$$\left[{\mathrm{Zn}}_{\mathrm{NTA}}\right]=\left[{\mathrm{NTA}}_{\mathrm{Zn}}\right]$$

$$\left[\mathrm{TLR}2_{\text{free}}\right]\times \left[{\mathrm{Zn}}_{\text{free}}\right]/\left[\mathrm{TLR}2_{\mathrm{Zn}}\right]={\mathrm{kd}}_{\mathrm{TLR}2_{\mathrm{Zn}}}$$

$$\left[{\mathrm{NTA}}_{\text{free}}\right]\times \left[{\mathrm{Zn}}_{\text{free}}\right]/\left[{\mathrm{NTA}}_{\mathrm{Zn}}\right]={\mathrm{kd}}_{{\mathrm{NTA}}_{\mathrm{Zn}}}$$

$${\mathrm{kd}}_{{\mathrm{NTA}}_{\mathrm{Zn}}}=4.4\;\mathrm{nM},$$

where NTA‐Zn binding constant was taken from the work [42]. Statistical analysis of the obtained constants was carried out in python v3.0 software (Python Software Foundation, Beaverton, OR, USA) using the open access libraries (Pandas, NumPy, SciPy). The *P*‐value was obtained from the calculated *t*‐statistic comparing two parameters with nonequilibrium data samples. The number of degrees of freedom of the compared elements is taken as the sum of the number of measurements of the compared elements minus the restriction on the degrees of freedom. The restrictions on the degrees of freedom were taken to be equal to three, which is the number of independent variables in the solved system of equations. The number of measurements of the compared value is taken as the number of experimental points used to calculate the dissociation constant.

To analyze the interaction between TLR1_TIR_ and TLR2_TIR_, we correlated the intensities of the NH cross‐peaks in the ^1^H,^15^N‐HSQC spectra recorded with and without the addition of TLR2_TIR_. The intensities were normalized to the intensity of a TLR1_TIR_ C‐terminal residue signal in the spectra, which is necessary to eliminate the uncertainty in the concentrations of the interacting proteins. In the EDTA competition assay, these data were processed in the same way. For TLR2_TIR_, a similar dependency was built. In the zinc ion titration experiments of TLR1_TIR_ with and without the addition of TLR2_TIR_, we assessed the concentration of the apo state using the approach described above for TLR2_TIR_. Six TLR1_TIR_ NH‐signals were selected as separate narrow TLR1_TIR_ signals corresponding to the stable regions of the molecule and not overlapping with the TLR2_TIR_ NH‐signals. The *P*‐value was calculated using the method described above for the compared data samples of equal size. The number of analyzed signals was used as the number of independent repetitions. The restriction on the degrees of freedom was taken as standard (equal to two).

### Secreted embryonic alkaline phosphatase (SEAP) assay

HEK‐Blue Null2 cells, stably transfected with a SEAP reporter gene under the control of an NF‐κB transcriptional response element, were obtained from InvivoGen (San Diego, CA, USA, RRID: CVCL_A7YM). Authentication of the HEK‐Blue Null2 cells was carried out using the Short Tandem Repeat (STR) method in 2022. Prior to experiments, cells were tested for mycoplasma by a MycoBlue Mycoplasma Detector (Vazyme Biotech, Nanjing, China) to exclude contamination. HEK Blue Null2 cells (InvivoGen, hkb‐null2), stably transfected with a SEAP reporter gene under the control of an NF‐κB transcriptional response element, were cultured in Dulbecco's modified Eagle's medium supplemented with 10% fetal bovine serum (FBS), penicillin (50 unit·mL^−1^), streptomycin (50 μg·mL^−1^), and 1× HEK blue selection. HEK Blue Null2 cells were co‐transfected with human TLR1 or TLR6 and either wild‐type or mutant forms of human TLR2 using Lipofectamine 2000 (Invitrogen, Carlsbad, CA, USA) according to the manufacturer's instruction. After 48 h of transfection, the transfected cells were seeded at a density of 1 × 10^5^ cells·mL^−1^. After 24 h incubation, the medium was changed to Opti‐MEM medium supplemented with 0.5% FBS, penicillin (50 unit·mL^−1^), streptomycin (50 μg·mL^−1^), 1% of the nonessential amino acid (NEAA), and either Pam_3_CSK_4_ (50 ng·mL^−1^, for TLR1/TLR2) or Pam_2_CSK_4_ (50 ng·mL^−1^, for TRL6/TLR2) for 8 h. NF‐κB activity was detected by the Phospha‐Light™ SEAP Reporter Gene Assay System (Applied Biosystems, Foster, CA, USA) according to the manufacturer's instructions. All the experiments were repeated four times and analyzed using the directional Student's *t*‐test.

### Quantitative reverse‐transcriptase PCR (qRT‐PCR)

HEK Blue Null2 cells expressing human TLR1 or TLR6 and either wild‐type or mutant forms of human TLR2 were seeded at a density of 2 × 10^5^ cells·mL^−1^ in six‐well plates. After 24‐h incubation, total RNA was extracted using the RNeasy Mini Kit. cDNA was synthesized with the RT^2^ Easy First Strand cDNA Synthesis Kit. Quantitative PCR (qPCR) was performed on a TOptical Real‐Time qPCR Thermal Cycler (Analytik Jena, Thuringia, Germany) using the SYBR Green method. Data were analyzed by the ^ΔΔ^Ct method. All the experiments were repeated four times and analyzed using the directional Student's *t*‐test. The sequences of the primers used were as follows: β‐actin (forward: 5′‐TCGTGCGTGACATTAAGGAG‐3′, reverse: 5′‐ATGCCAGGGTACATGGTGGT‐3′), TLR1 (forward: 5′‐GCTGATCGTCACCATCGTTG‐3′, reverse: 5′‐GTCCACTGGCACACCATCCT‐3′), TLR2 (forward: 5′‐CCTCTCGGTGTCGGAATGTC‐3′, reverse: 5′‐GGCCCACATCATTTTCATATACC‐3′), and TLR6 (forward: 5′‐ATAGAGGAAGCCCACTAAAGGACT‐3′, reverse: 5′‐ACAGTCACAGCCAACACCAGC‐3′).

## Results

### TLR2_TIR_ binds the Zn ions with nanomolar affinity

**Fig. 1:**
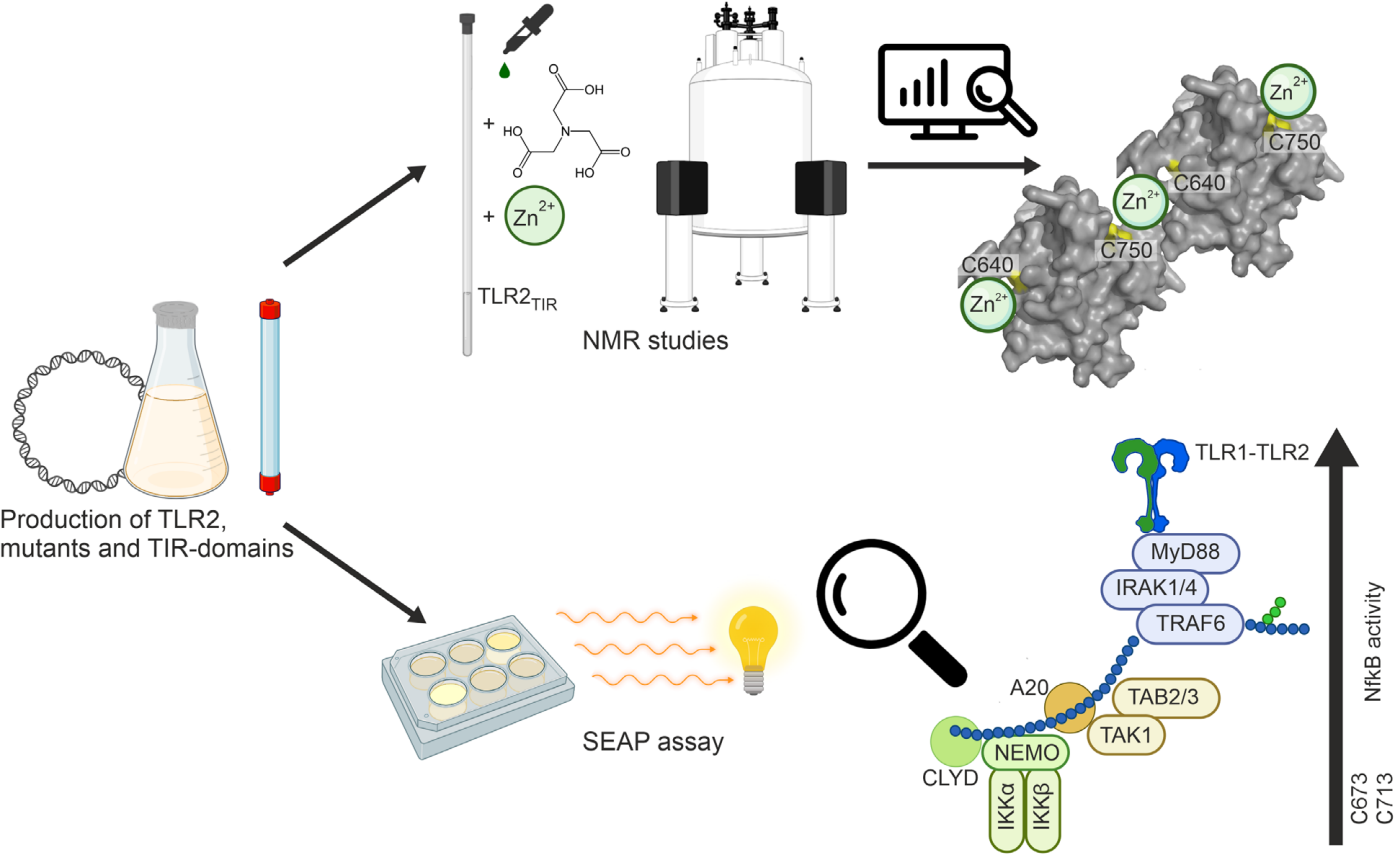
The overall scheme of the studies. The first step was to obtain the wild‐type and cysteine mutant variants of TLR2_TIR_. After that, the interaction of TLR2_TIR_ with zinc ions in the presence of NTA was studied using NMR for each sample, leading to hypotheses regarding the formation of the Zn‐TLR2_TIR_ complex. Functional assays were performed to assess the impact of cysteine mutations in TLR2 on the activity of the full‐length TLR1‐TLR2 and TLR6‐TLR2 receptors.

**Fig. 2:**
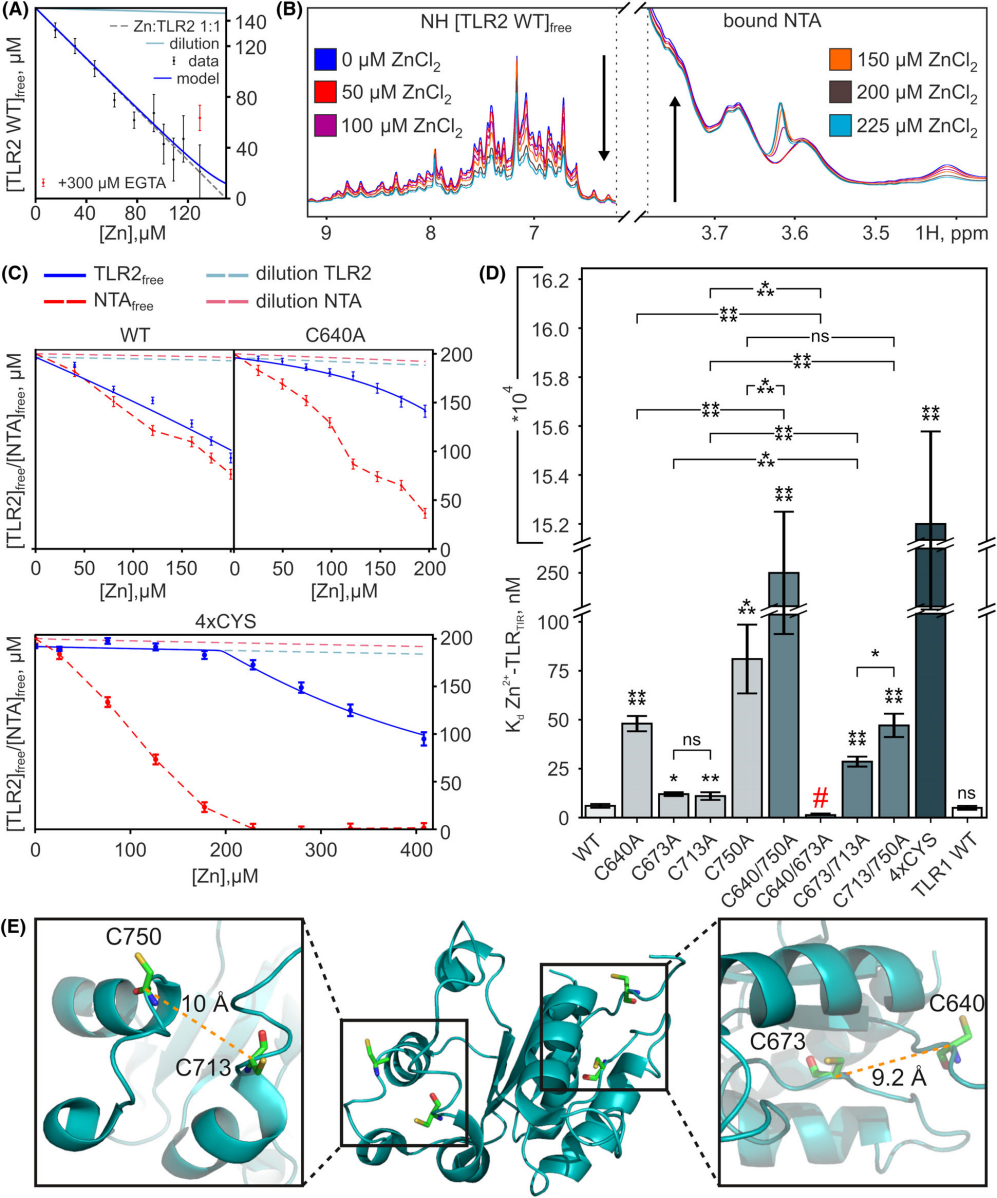
Analysis of the interaction of zinc with the WT and mutant TLR2_TIR_. (A) The analysis of signal intensity changes during the titration of TLR2_TIR_ WT with zinc. The concentration of free TLR2_TIR_ was obtained from the analysis of peak intensities in ^1^H,^15^N‐HSQC NMR spectra. (B) Fragments of ^1^H NMR spectra of WT TLR2_TIR_ obtained in the course of the competitive binding experiments. Regions of the spectra that change during Zn titration are presented: protein amide/aromatic signals (left) and the signal of Zn‐bound NTA (right). The direction of signal intensity changes upon zinc addition is indicated by black arrows. (C) The analysis of signal intensity changes during competitive binding experiments for WT, C640A, and 4xCYS mutants. Data for all the proteins considered in the work are presented in Fig. S3. The concentration of free TLR2_TIR_ was obtained from the analysis of peak intensities in ^1^H,^15^N‐HSQC NMR spectra. The measurement error was determined from the average value of the analyzed peaks and the signal‐to‐noise ratio. (D) The *K*_d_ values obtained for the Zn: TLR2_TIR_ complex. The red # indicates an untrustworthy value of the measured constant due to the instability of the C640/673A protein. Error bars indicate the standard deviations (measurement error). Statistical analysis was carried out based on the calculation of *t*‐statistics with the further determination of the *P*‐value (*P < 0.05, **P < 0.01, ***P < 0.001, ****P < 0.0001, ns denotes that changes are not significant with respect to the WT or indicated pair). (E) The spatial structure of TLR2_TIR_ in the ribbon representation. The structure is based on PDB 1FYW [22]. Cysteines are shown with sticks.

Since the previous data on Zn binding to TLR1 [31] were obtained by NMR spectroscopy, we chose the same approach to investigate TLR2 (Fig. 1). For this purpose, we synthesized the ^15^N‐labeled TLR2_TIR_ [39] and titrated it with Zn ions. Only one set of signals was observed in the ^15^N‐HSQC spectrum of TLR2_TIR_ without chemical shift perturbations (Fig. 2A and Fig. S1). At the same time, the intensities of all signals in the NMR spectra were uniformly reduced upon the addition of Zn (Fig. S1). The decrease in signal intensity during titration clearly indicates the formation of oligomeric complexes in the presence of Zn ions, as previously observed [43]. The intensity of the NH signals of TLR2_TIR_ decays with a slope close to one without significant deviations. This indicates that Zn binds to the protein at a 1 : 1 stoichiometry, with the dissociation constant (*K*_d_) that is much lower than the protein and Zn concentrations, preventing its determination. Therefore, we utilized a competitive binding assay to estimate the *K*_d_ of the Zn : TLR2_TIR_ complex. The ^15^N‐labeled TLR2_TIR_ was mixed with NTA (1 : 1, mole : mole), titrated with Zn, and the average decrease of protein signal intensities in 2D NMR spectra was analyzed together with the intensity of the peak corresponding to the Zn‐bound NTA in 1D ^1^H NMR spectra (Fig. 2B,C). In addition to NTA, EDTA and EGTA were tested as possible alternatives, but TLR2_TIR_ was unable to compete with them for the Zn ions, implying that the Zn‐binding affinity of TLR2 is weaker than that of EGTA (0.45 nm) but comparable to that of NTA (4.4 nm). The resulting *K*_d_ (Zn : TLR2_TIR_) was 6 ± 1 nm (Fig. 2С and Fig. S3), and the application of the same approach to the TLR1_TIR_ protein yielded *K*_d_ = 5 ± 2 nm, consistent with 4.5 ± 0.5 nm previously measured by the EGTA competition assay [31] (Fig. S3).

To verify this result with an orthogonal approach, we performed SEC and DLS analysis. Zinc was added to the TLR2_TIR_ at various concentrations; the sample was centrifuged to remove the aggregates, and the solution was injected into the SEC column. Only one form was detected in all samples; however, the amount of protein in the peak was diminished upon each Zn addition (Fig. S4). Furthermore, no substantial shift of the main peak was observed. The elution volume for all samples corresponds to the protein with a molecular weight of ~ 25 kDa, which is very close to a monomeric form (19.9 kDa). DLS analysis of this peak confirms that the protein species have a MW of ~ 19 kDa. The decrease in protein concentration is in good agreement with the NMR data: approximately 50% of the protein remains in solution at a Zn : protein ratio of 0.5 : 1 (Fig. 2A and Fig. S4A).

To conclude, TLR2_TIR_ binds Zn ions with nanomolar affinity at a 1 : 1 stoichiometry, almost identical to the affinity of TLR1_TIR_. This interaction leads to high‐order oligomerization and precipitation of the protein.

### Cysteines of TLR2_TIR_ play a key role in Zn binding

Since cysteines are the most abundant residues in the coordinating spheres of zinc ions [44] and are responsible for Zn binding in TLR1_TIR_ [31], we first performed the cysteine scanning mutagenesis studies to identify the key residues for the Zn binding activity of TLR2_TIR_. The protein has four cysteines, only two of which are conserved in the TLR1/2/6/10 subfamily: 673 and 713 (Fig. S5). We designed a mutant with all four cysteines replaced by alanines (4xCYS) and applied the same competitive binding assay. No decrease in signal intensities was detected during titration up to a Zn : TLR2_TIR_ ratio of 1 : 1 (mole : mole), while all added Zn bound to NTA (Fig. 2C). The obtained *K*_d_ was equal to 152 ± 13 μm, indicating an almost complete loss of specific zinc binding propensity and an essential role of cysteines in the interactions of TLR2 with Zn ions.

In this regard, we next investigated four single‐point mutants (C640A, C673A, C713A, and C750A). To assess the influence of the mutations on the protein spatial structure, the ^15^N‐HSQC spectra of the mutants were compared with those of the wild‐type protein (WT), and in all cases, no significant changes were observed; most peaks remained at their positions, indicating that the global fold is maintained. Several peaks disappeared and new signals appeared, confirming the introduction of the mutations into the protein (Figs S6–S9). The *K*_d_ values obtained for all samples are shown in Table 1 and Fig. 2D. Although each mutation led to a lower affinity, it still remained in the nanomolar range, suggesting the presence of at least two or more binding sites: blocking one site slightly reduces the binding affinity, but Zn binding persists. At the same time, the *K*_d_ for C640A and C750A samples increased by an order of magnitude or more (Fig. 2C,D). In the 3D structure of TLR2 [22] these cysteines are located on the opposite faces of the protein molecule (Fig. 2E), suggesting that Zn ions bind simultaneously to different sites on different TLR2 molecules, causing asymmetric oligomerization. Only in this case can a single mutation decrease Zn affinity in the presence of multiple binding sites. In other words, the Zn binding sites are formed at the interfaces of the TLR2/TLR2 contacts, with Zn acting as a bridging ion.

**Table 1:** The results of competitive binding experiments for the wild‐type (WT) and cysteine mutants of TLR2_TIR_.

Protein	*K*_d_, nm	Protein	*K*_d_, nm	Protein	*K*_d_, nm
TLR1 WT	5 ± 2	C640A	48 ± 4	C640/673A	1.5 ± 0.5
WT	6 ± 1	C673A	12 ± 1	C640/750A	252 ± 32
4xCYS	(152 ± 13) × 10^3^	C713A	11 ± 2	C673/713A	29 ± 3
		C750A	81 ± 18	C713/750A	41 ± 6

These data do not provide a clear understanding of zinc binding since each single‐point mutation affects the protein affinity. Therefore, we proceeded with double mutants. In the 3D structure of TLR2, the cysteines are paired: C673 is close to C640, while C713 is a neighbor of C750 (Fig. 2E). Therefore, we designed 4 additional mutants: C640/750A (two cysteines with strong effect on Zn‐binding), C673/713A (two cysteines with weak effect), C640/673A, and C713/750A (cysteines in each pair are proximal in the 3D structure). Comparison of the ^15^N‐HSQC spectra of the double mutants with the WT showed minor changes for all protein variants, except for the C640/673A sample (Figs S10–S13). In the latter case, 10 new signals are present, whereas no more than three extra signals appeared in the spectra of the other mutants. We believe that this indicates local rearrangements within the protein. Nevertheless, competitive binding experiments were carried out for the samples. The greatest reduction in protein affinity was observed for C640/750A (252 ± 32 nm), whose *K*_d_ was about 40‐fold weaker than that of WT, but at the same time three orders of magnitude lower than that of the all‐cysteine mutant 4xCYS (152 μm) (Fig. 2D). In contrast, the *K*_d_ for C640/673A (1.5 ± 0.5 nm) was similar to that of WT, although this value is not entirely reliable due to the low stability of this particular mutant and its tendency to precipitate. Interestingly, the 673/713A substitution also caused a significant effect (5‐fold increase in *K*_d_ compared to WT), suggesting that these two residues are somehow involved in Zn binding. At the same time, 713/750A bound zinc better than 750A. SEC analysis for the C640A and C713A/C750A mutants shows the same pattern (Fig. S4D,E). Equimolar addition of zinc results in reduced oligomerization and precipitation of the mutant forms compared to WT, in perfect agreement with our NMR data.

To exclude the possibility that the observed process is a zinc‐induced irreversible precipitation of TLR2 TIR, we performed two additional experiments. First, we added an equimolar mixture of NTA and zinc to the protein instead of adding zinc alone to the protein:NTA mixture. As shown in Fig. S14, the decrease in signal intensities is clearly observed in all cases, especially for WT and C713A mutant. Moreover, the Zn binding affinities obtained in this experiment are very close to those measured above: 6.3 ± 0.7 nm (now) vs. 6 ± 1 nm (above) for WT, 11 ± 2 nm vs. 12.6 ± 0.2 nm for C713A, and 265 ± 10 nm vs. 252 ± 32 nm for C640/750A. This shows that TLR2‐TIR is able to bind Zn even when it is already bound to NTA and further confirms the presence of competition. Second, we performed the EDTA competition assay. As shown in Fig. S15, when EDTA is added after 1.5 h of protein incubation with zinc, the protein is recovered to 85% of the initial concentration. However, when EDTA is added after 24 h, only 50% of the protein can be recovered. We speculate that some aggregates become larger and more compact over time, making it difficult for EDTA to penetrate deeply into them. Thus, the results obtained confirm that the interaction with zinc ions leads to the reversible oligomerization of TLR2 TIR, with a fraction of the protein (up to 50%) aggregating irreversibly after a certain period of time.

To summarize, although C640 and C750 show a greater effect on Zn binding, all four cysteines are involved in the observed process. Zn is a bridging ion that brings together two faces of TLR2_TIR_ monomers, causing protein oligomerization and precipitation. Multiple modes of Zn binding are possible, explaining the rather subtle effects of some single‐point mutants. Apparently, the structure of the resulting oligomer can adapt to the absence of 1–2 cysteine residues, providing a variety of possible arrangements of high‐order oligomers. In support of this hypothesis, it is important to note that the presence of multiple binding sites is likely to lead to high‐order oligomerization and precipitation, as observed in NMR and SEC experiments.

### TIR domains of TLR1 and TLR2 do not interact with each other but compete for Zn

**Fig. 3:**
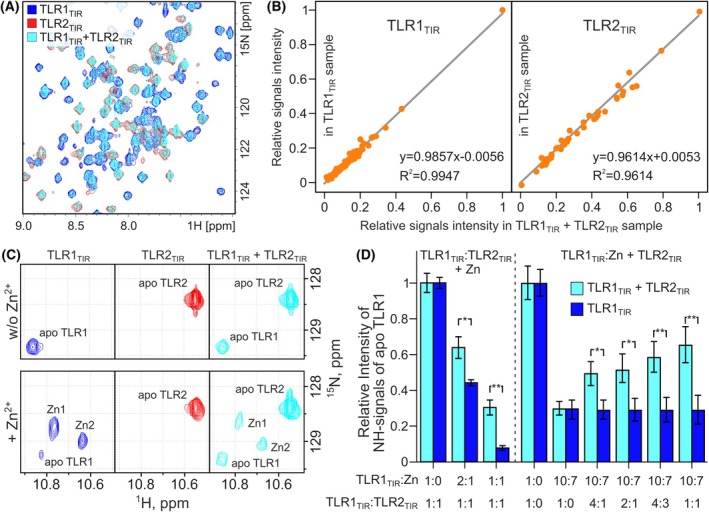
TLR1_TIR_ and TLR2_TIR_ compete for the zinc. (A) Overlay of ^1^H,^15^N‐HSQC spectra of TLR1_TIR_ (blue), TLR2_TIR_ (red), and equimolar mix of TLR1_TIR_ and TLR2_TIR_ (cyan) (100 μm each). (В) Comparison of signal intensities of the TLR1_TIR_ taken separately and in the equimolar mix of TLR1_TIR_/TLR2_TIR_ (left) and TLR2_TIR_ taken separately and in the equimolar mix of TLR1_TIR_/TLR2_TIR_ (right). (C) Fragments of ^1^H,^15^N‐HSQC NMR spectra of TLR1_TIR_, TLR2_TIR_, and their equimolar mix with and without Zn. (D) Signal intensities of apo TLR1_TIR_ (100 μm) with and without the TLR2_TIR_ (100 μm) upon addition of Zn (left, before vertical dotted line) and with and without the Zn upon addition of TLR2_TIR_ (right). Error bars indicate the standard deviations. Statistical analysis was carried out based on the calculation of *t*‐statistics with the further determination of the *P*‐value (*P < 0.05, **P < 0.01).

Since TLR2 acts as a heterodimer with either TLR1 or TLR6, we tested the hypothesis of receptor activation by the interaction of the TIR domains of these proteins. It turned out that the addition of TLR2_TIR_ to TLR1_TIR_ had no effect on the NMR spectra—no changes in chemical shifts and cross‐peak intensities were detected for either TLR1_TIR_ or TLR2_TIR_ (Fig. 3A, and Figs S16 and S17). Analysis of the signal intensities revealed that the correlation between the signal intensities of TLR1_TIR_ taken separately and TLR1_TIR_ taken in a mixture with TLR2_TIR_ was 0.9857, and the Pearson coefficient was 0.9947 (Fig. 3B). A similar analysis for TLR2_TIR_ provides the correlation coefficient and Pearson coefficient of 0.9614. The results obtained confirm the identity of the compared NMR spectra and the absence of interaction. This may indicate the presence of additional participants in the formation of the TLR1_TIR_/TLR2_TIR_ complex, such as the metal ions.

Since TLR1_TIR_ and TLR2_TIR_ were shown to form complexes with zinc ions with nanomolar affinity, we studied whether TLR1_TIR_ and TLR2_TIR_ can interact in the presence of Zn (Fig. 3C,D). The addition of Zn to the mixture of TLR1_TIR_ and TLR2_TIR_ did not alter the behavior of the proteins analyzed. In the case of TLR1_TIR_, two additional sets of signals appeared, and in the case of TLR2_TIR_, we observed a gradual decrease in signal intensity, similar to the proteins studied separately. At the same time, TLR1_TIR_ and TLR2_TIR_ competed for Zn (Fig. 3D): in the case of TLR1_TIR_, an increased content of the apo form of the protein was observed compared to a similar experiment without the addition of TLR2_TIR_. Similar results were obtained when TLR2_TIR_ was added to a preformed TLR1_TIR_ : zinc complex (Fig. 3D). These data suggest that TLR1_TIR_ is not recruited to the Zn‐induced oligomers of TLR2_TIR_. Thus, we can state that no interaction between the TIR domains of TLR1 and TLR2 was observed even in the presence of Zn ions, suggesting that either some additional factor is missing in our model system, or that the direct TIR/TIR interaction in TLRs is not required for TLR signaling.

### C673 and C713 are important for TLR2 signaling

**Fig. 4:**
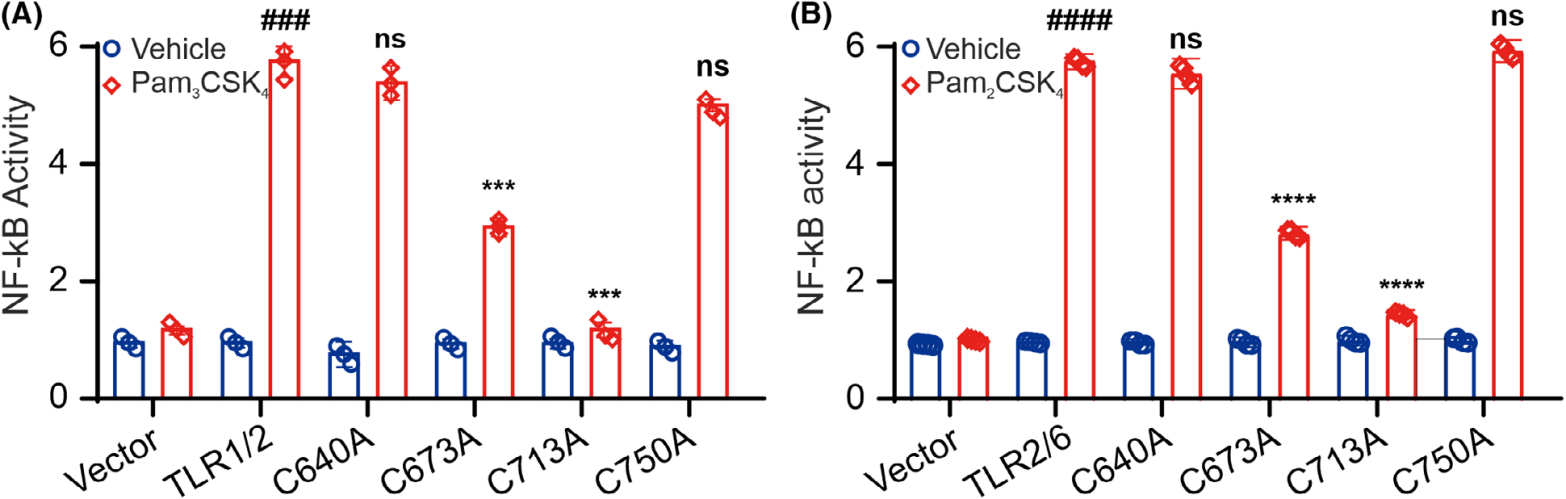
The role of cysteines in the activation of TLR2. Effect of cysteine mutations of TLR2 in TLR1/2 (A) and TLR2/6 (B) receptor activation. NF‐κB activity was measured for TLR2 mutants upon stimulation with Pam_3_CSK_4_ or Pam_2_CSK_4_, respectively (*n* = 4 independent experiments). Statistical significance is indicated as follows: ***P < 0.001 and ****P < 0.0001 with respect to the positive control, ###*P* < 0.001, ####*P* < 0.0001 with respect to the negative control experiments. ns denotes that changes with respect to the positive control are not significant. Error bars represent the standard error of the mean.

To investigate the role of cysteines in TLR2 activity, a functional assay was performed. First, HEK Blue Null2 cells were cotransfected with TLR1 and either wild‐type TLR2 or its single‐point cysteine mutants. The TLR1/2 receptor complex was then stimulated with its specific ligand, Pam_3_CSK_4_, and nuclear factor‐κB (NF‐κB) activity was monitored using the Phospha‐Light secreted embryonic alkaline phosphatase (SEAP) reporter gene assay system. Surprisingly, only two cysteine mutants, C673A and C713A, showed a significant decrease in receptor activity (Fig. 4A). Cysteines C640 and C750, which had the most pronounced effect on Zn binding to the TLR2_TIR_ domain, did not affect NF‐κB activation. To test whether these cysteines could be involved in the other TLR2 heterodimer, we performed the same tests on the TLR6/2 receptor complex. Cells were co‐transfected with TLR6 and either wild‐type TLR2 or its single‐point cysteine mutants, and the TLR6/2 receptor complex was stimulated with its specific ligand, Pam_2_CSK_4_. The results were almost identical to those observed with TLR1/2 (Fig. 4B). The expression levels of TLR1, TLR2, TLR6, and all single‐point cysteine mutants of TLR2 were comparable (Fig. S18). The fact that both TLR2/1 and TLR2/6 signaling share the same critical cysteine residues suggests that these residues are essential for homomeric interactions or binding of downstream adaptor proteins rather than for heterodimerization.

Thus, we can conclude that C673 and C713 are the key residues for TLR2‐based receptor activation and downstream signaling.

### Zn binding sites

**Fig. 5:**
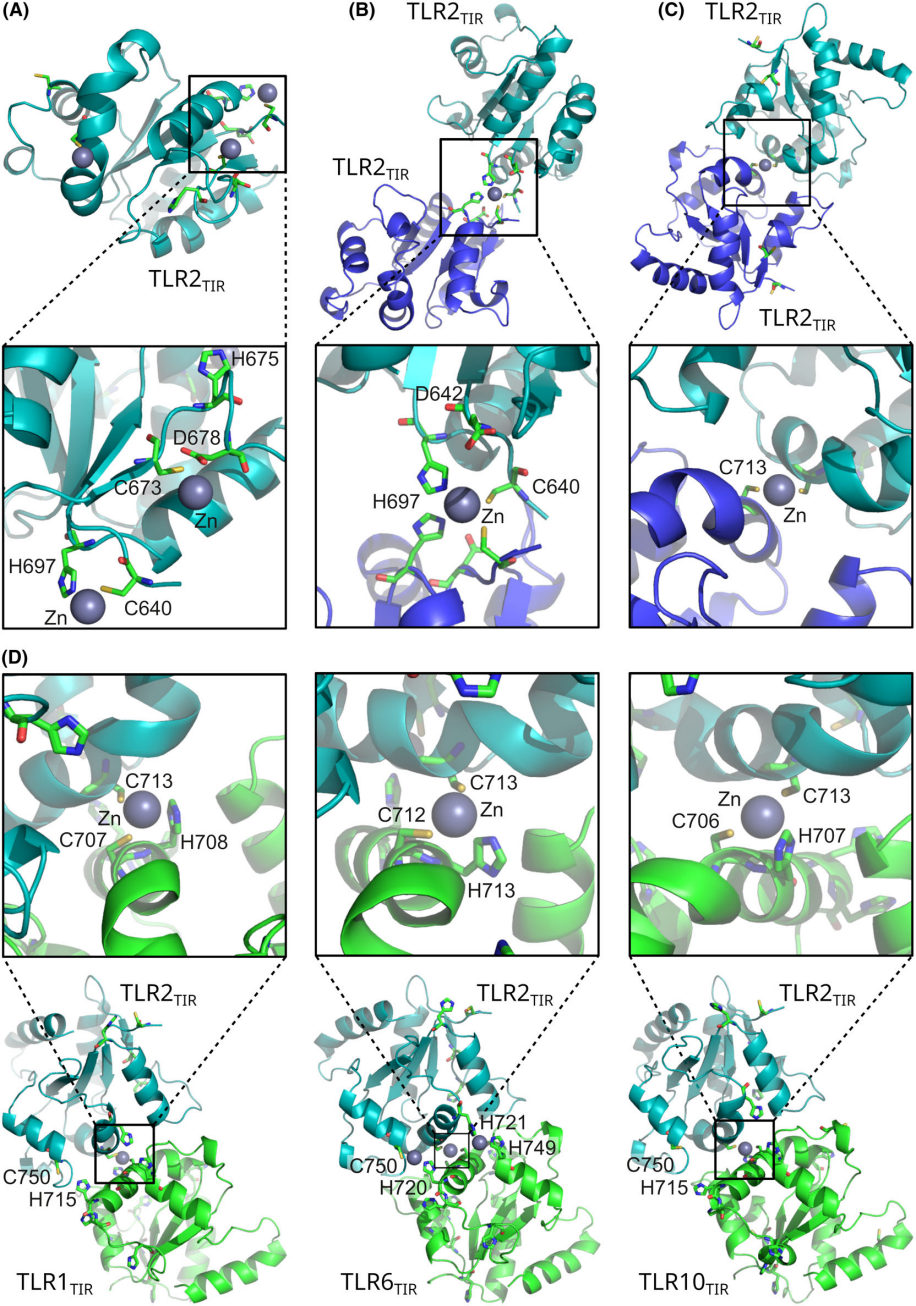
Zn binding sites prediction. (A) The prediction of Zn binding sites for monomer TLR2_TIR_ (A), homodimer TLR2_TIR_:TLR2_TIR_ (B, C), and heterodimers TLR2_TIR_:TLR1_TIR_, TLR2_TIR_:TLR6_TIR_, TLR2_TIR_:TLR10_TIR_ (D). All predictions were generated by alphafold 3 [45], except for panel (B), which was constructed manually.

To understand the structural basis of the Zn : TLR2_TIR_ interaction, we examined the local environment of TLR2_TIR_ cysteines on the protein structure and used alphafold 3 software [45] to predict the Zn binding sites. Three sites involving C640, C673, and C713 were identified for the monomeric TLR2_TIR_ (Fig. 5A). Interaction between Zn and C640 was observed in all models, and Zn was coordinated by C640 and H697 side chains. Considering the classical CCHH zinc finger motif, it is possible that Zn is coordinated by two TIR domains, which is in agreement with our NMR data (Fig. 5B). Two other sites were predicted in several TLR2_TIR_ monomer models and included C673/D678 and C713, respectively (Fig. 5A). The queries with two TLR2_TIR_ molecules showed a dimer with two C713 coordinating a Zn ion (Fig. 5C). Furthermore, С713 is involved in all possible variants of the heterodimeric TIR:TIR complexes (TLR1/2, TLR6/2, TLR10/2), where the coordination occurs *via* C713 of TLR2 and C707/H708 of TLR1, C712/H713 of TLR6, and C706/H707 of TLR10 (Fig. 5D). While we did not find any models with Zn:C750 interactions, this cysteine is close to C713 and histidine of partner receptors: H715 of TLR1 or TLR10 and H720 of TLR6 (Fig. 5D).

While alphafold provides several Zn‐binding modes of TLR2_TIR_ in homo‐ and heterodimers, other possible Zn coordination modes are likely not detected by this software. Besides, the heterodimer models resemble the X‐ray structures with a disulfide bond between C713. Nevertheless, these results suggest that various modes of zinc binding involving different cysteines are possible, which may explain the protein aggregation in our study and promote interactions with adaptor proteins.

## Discussion

Previously, we demonstrated the propensity of the TIR domain of human TLR1 to specifically bind zinc ions with nanomolar affinity [31]. An in‐depth investigation revealed the important role of cysteines in Zn interaction and receptor activation. The protein sequence alignment clearly shows that two cysteines are conserved for the TIR domains of TLR (Fig. S5). Indeed, C673 (TLR2 notation) is present in all TLR members and is located in the BB‐loop (Fig. 2E and Fig. S5). Although this region is disordered, many studies show its essential role in the interaction with adaptor proteins [46]. We found that this cysteine in TLR1 (C667) is most important for Zn binding and is a key residue for receptor signaling [31]. C713 (TLR2 notation) is present in most TLRs, but is absent in the TLR7/8/9 branch (Fig. S5). We did not find any relationship between this cysteine and receptor activation for either TLR1 or TLR2 in the literature. Other cysteines show no conservation and are randomly distributed throughout the TIR sequences. At the same time, we showed that C686 in TLR1 is also involved in Zn coordination: one of the two Zn‐binding sites is formed by C667 and C686 [31].

Based on these data and considering the heteromeric nature of the TLR1/2 receptor complex, we hypothesized that TLR2 could bind zinc ions like TLR1. Here we present several findings: (a) the TIR domain of TLR2 is able to bind Zn with nanomolar affinity; (b) all cysteines of the TLR2_TIR_ are important for these interactions; (c) two of them, C673 and C713, are essential for receptor activation. The affinities of TLR1_TIR_ and TLR2_TIR_ for Zn are close to each other: 5 ± 2 and 6 ± 1 nm, respectively. We suggest that these values are biologically relevant. A large number of proteins interact with Zn [32] and their affinity can be even micromolar [42, 47, 48, 49]. Several zinc‐finger proteins have a nanomolar constant or even weaker affinity [47]. Moreover, a rapid increase in intracellular zinc ion concentration, known as the ‘zinc wave’, can occur upon extracellular stimulation [33]. Thus, the Zn affinity of TLR1 and TLR2 TIR domains is significant, and they may be involved in intracellular zinc cascades.

For both TLR1 and TLR2, cysteines are the key participants in Zn binding; however, it is challenging to elucidate the specific roles of individual residues. Only two of the three cysteines in TLR1 are involved in Zn binding, whereas the BB‐loop cysteine is essential. At the same time, all four cysteines in TLR2 interact with Zn ions and show a cooperative effect. In contrast to TLR1, the most significant outcome is obtained by mutating the N‐ and C‐terminal residues (640 and 750), which are not conserved in the TLR family (Fig. S5). Moreover, they are not important for NF‐κB signaling according to our functional assay, indicating a possible inconsistency. This discrepancy could be resolved by analyzing the obtained data. First, we observed that zinc acts as a bridging ion, most likely binding at the interface of interacting TIR domains and stimulating their oligomerization and precipitation. In the case of TLR2_TIR_, zinc binds to C640 of one TIR and C750 of another TIR, and this binding site could be formed only in the context of asymmetric TLR2_TIR_ homodimerization. These residues are located on opposite sides of the protein, but the mutation of each of these amino acids significantly reduces oligomerization/precipitation. Apparently, zinc‐induced oligomerization of TLR2 plays no role in the initiation of the downstream NF‐κB cascade, as the mutations of C640 and 750 show no inhibitory effect. On the other hand, these cysteines could be used for processes other than activation. It is known that zinc supplementation downregulates inflammation, and several zinc‐finger domain‐containing proteins (A20, ZBTB20, ZCCHC11, etc.) involved in TLR‐signaling pathways are modulators of inflammation [50, 51, 52, 53, 54, 55]. Moreover, APIN1, an A20‐binding inhibitor, regulates the immune response through the C/EBPβ pathway without affecting the NF‐κB and MAPK cascades [56]. In other words, there are many ways in which these two residues and their zinc binding ability might be involved in TLR signaling that would not be detected in our functional assays. Thus, zinc binding to residues C640 and C750 could be a possible way to control the alternative TLR signaling pathways.

In contrast, in the presence of only two cysteines, C673 and C713 (C640/750 mutant), a significant weakening of zinc binding and less protein precipitation was observed. These residues may be critical for bridging between TLR2 and its adapter proteins TIRAP/MAL and MyD88. It is unlikely that these residues take part in the interactions between the TIR domains of TLR2 and their partner TLRs, TLR1, and TLR6, for two reasons: the same cysteines are essential for both TLR1/2 and TLR6/2 activation, and no interactions between the TLR1 and TLR2 TIR domains are observed in the presence of Zn in our *in vitro* experiments.

On the other hand, interaction with zinc leads to rapid high‐order oligomerization of protein‐zinc complexes and their precipitation. This may explain why we do not observe interactions between TLR1 and TLR2 in the presence of zinc. If such interactions do occur, another process should be considered: the formation of disulfide bonds. First, while the original structure of the TLR2 TIR domain (1FYW) is monomeric, there is a structure of tetrameric disulfide cross‐linked TLR2 (1O77). Second, a study by Lee *et al*. [43] demonstrated variants of homo‐ and heterodimer formation for TLR1 and TLR2, even in the presence of a reducing agent. Cross‐linking mass‐spectrometry experiments identified different modes of TLR1/TLR2 heterodimer formation involving the disulfide bonds. The authors proposed that despite the reducing conditions inside the cell, the formation of disulfide complexes is possible through a ‘dock‐and‐lock’ mechanism. It is noteworthy that we also observed the formation of disulfide dimers upon freezing and thawing of the sample, prolonged incubation, or low concentrations of the reducing agent in the sample. If such processes occur inside the cell, zinc may act as a catalyst for these reactions. Ligand binding could bring the TIR domains closer together, facilitating the rapid formation of a disulfide bond in the presence of zinc. Although the alphafold data cannot be considered as experimental evidence, our models show that the presence of zinc brings cysteines closer together (Fig. 5), potentially leading to their cross‐linking. Following the hypothesis proposed by Lee *et al*., such receptor homodimerization may serve as an ‘inhibitor’ of inflammation when TLR expression on immune cells is high, thereby protecting the cell from damage. In contrast, heterodimerization may act as a pro‐inflammatory signal, initiating the assembly of signaling complexes.

Finally, neither single nor double mutations of C673/713 alter the structure of TLR2_TIR_, suggesting that the effects of these substitutions are not folding‐related. This leaves us only with the possibility that these residues are involved in the zinc‐mediated protein–protein interactions with the adaptors and thus in the assembly of supramolecular signaling complexes (Myddosomes) [57]. Recently, Fisch *et al*. [58] showed that TLRs induce Myddosome assembly, and the size of the Myddosome varies up to the micron level (> 2 μm^2^), implying the important role played by oligomerization. Since in our hands Zn acts as a bridging ion, inducing the oligomerization of TIR domains, the involvement of Zn and cysteines 673/713 in the initial stages of signalosome assembly seems plausible, although this hypothesis requires further verification. Here, we confidently state that an interaction between TLR2 and Zn occurs *in vitro*, leading to oligomerization and precipitation. Cysteines play a key role in this process. While we also demonstrate the involvement of two of the four cysteines in signaling processes *in vivo*, the mechanism of TLR activation and its relationship to the Zn‐bound states of the receptor remain unclear. Further *in vivo* studies are needed to unravel the complex relationships between Zn and TLRs.

## Author contributions

Experiments: VAL, CL, IAT, MVG. Analyzing and interpreting: VAL, SAG, KSM. Supervising: XW, SAG, KSM. Funding and resources: ASA, EVB. Writing (original draft): VAL, SAG. Writing (editing): VAL, SAG, XW, KSM.

## Peer review

The peer review history for this article is available at https://www.webofscience.com/api/gateway/wos/peer‐review/10.1002/1873‐3468.70026.

## Supporting information


**Fig. S1.** The effect of adding zinc to TLR2_TIR_.
**Fig. S2.** Determination of the average rate of TLR2_TIR_ oligomerization.
**Fig. S3.** Titration of WT TLR1_TIR_, WT TLR2_TIR_ and its mutants with Zn in the presence of NTA.
**Fig. S4.** Analysis of TLR2_TIR_ samples.
**Fig. S5.** Alignment of protein sequences of all the human TLR TIR.
**Fig. S6.** Superposition of ^1^H,^15^N‐HSQC spectra of WT TLR2_TIR_ and TLR2_TIR_ C640A.
**Fig. S7.** Superposition of ^1^H,^15^N‐HSQC spectra of WT TLR2_TIR_ and TLR2_TIR_ C673A.
**Fig. S8.** Superposition of ^1^H,^15^N‐HSQC spectra of WT TLR2_TIR_ and TLR2_TIR_ C713A.
**Fig. S9.** Superposition of ^1^H,^15^N‐HSQC spectra of WT TLR2_TIR_ and TLR2_TIR_ C750A.
**Fig. S10.** Superposition of ^1^H,^15^N‐HSQC spectra of WT TLR2_TIR_ and TLR2_TIR_ C640/750A.
**Fig. S11.** Superposition of ^1^H,^15^N‐HSQC spectra of WT TLR2_TIR_ and TLR2_TIR_ C640/673A.
**Fig. S12.** Superposition of ^1^H,^15^N‐HSQC spectra of WT TLR2_TIR_ and TLR2_TIR_ C673/713A.
**Fig. S13.** Superposition of ^1^H,^15^N‐HSQC spectra of WT TLR2_TIR_ and TLR2_TIR_ C713/750A.
**Fig. S14.** The analysis of signal intensity changes in the competitive binding experiments for WT, C713 and C640/750A mutants.
**Fig. S15.** EDTA competition assay.
**Fig. S16.** Analysis of interaction between TLR1_TIR_ and TLR2_TIR_.
**Fig. S17.** Comparison of TLR1_TIR_/TLR2_TIR_ signal intensities in ^1^H,^15^N‐HSQC spectra before and after protein mixing.
**Fig. S18.** Expression levels of TLR2 and 6.
**Table S1.** PCR primers for WT TLR2_TIR_ and cysteines mutants.
**Table S2.** Gene constructs for expression of WT and mutants of TLR2_TIR_.
**Table S3.** Amino acid sequence of WT and mutants of TLR2_TIR_.

## Data Availability

The data that support the findings of this study are available in the Supporting Information and from the corresponding author ms.goncharuk@gmail.com or mineev@nmr.uni-frankfurt.de upon request.
