# Supplementary material for: The intracellular domain of TLR2 is capable of high‐affinity Zn binding: possible outcomes for the receptor activation
Source: FEBS Lett. 2025 Mar 3;599(13):1864–79. doi: 10.1002/1873-3468.70026 (PMC12258416; doi:10.1002/1873-3468.70026)
Supplement: Supplementary file 1 — Fig. S1. The effect of adding zinc to TLR2TIR. Fig. S2. Determination of the average rate of TLR2TIR oligomerization. Fig. S3. Titration of WT TLR1TIR, WT TLR2TIR and its mutants with Zn in the presence of NTA. Fig. S4. Analysis of TLR2TIR samples. Fig. S5. Alignment of protein sequences of all the human TLR TIR. Fig. S6. Superposition of 1H,15N‐HSQC spectra of WT TLR2TIR and TLR2TIR C640A. Fig. S7. Superposition of 1H,15N‐HSQC spectra of WT TLR2TIR and TLR2TIR C673A. Fig. S8. Superposition of 1H,15N‐HSQC spectra of WT TLR2TIR and TLR2TIR C713A. Fig. S9. Superposition of 1H,15N‐HSQC spectra of WT TLR2TIR and TLR2TIR C750A. Fig. S10. Superposition of 1H,15N‐HSQC spectra of WT TLR2TIR and TLR2TIR C640/750A. Fig. S11. Superposition of 1H,15N‐HSQC spectra of WT TLR2TIR and TLR2TIR C640/673A. Fig. S12. Superposition of 1H,15N‐HSQC spectra of WT TLR2TIR and TLR2TIR C673/713A. Fig. S13. Superposition of 1H,15N‐HSQC spectra of WT TLR2TIR and TLR2TIR C713/750A. Fig. S14. The analysis of signal intensity changes in the competitive binding experiments for WT, C713 and C640/750A mutants. Fig. S15. EDTA competition assay. Fig. S16. Analysis of interaction between TLR1TIR and TLR2TIR. Fig. S17. Comparison of TLR1TIR/TLR2TIR signal intensities in 1H,15N‐HSQC spectra before and after protein mixing. Fig. S18. Expression levels of TLR2 and 6. Table S1. PCR primers for WT TLR2TIR and cysteines mutants. Table S2. Gene constructs for expression of WT and mutants of TLR2TIR. Table S3. Amino acid sequence of WT and mutants of TLR2TIR. [file FEB2-599-1864-s001.docx]

Supplementary materials

The intracellular domain of TLR2 is capable of high-affinity Zn binding: possible outcomes for the receptor activation

Vladislav A. Lushpa, Cong Lin, Irina A. Talyzina, Marina V. Goncharuk, Eduard V. Bocharov, Alexander S. Arseniev, Xiaohui Wang, Sergey A. Goncharuk, Konstantin S. Mineev

**Table S1. PCR primers for WT TLR2_TIR_ and cysteines mutants.**

| protein | Primers (5’ → 3’) | |
| --- | --- | --- |
| WT TLR2_TIR_ | forward | ATATAGGATCCAGCCGTAAGATTTGTTACGAC |
|  | reverse | TATCAAGCTTATCACGATTTAATAG |
| C640A | forward | GGATCCAGCCGTAAGATTGCTTACGACGCATT |
|  | reverse | TATCAAGCTTATCACGATTTAATAG |
| C673A | forward | TTAAACTGGCCCTGCATAAACG |
|  | reverse | ATGCAGGGCCAGTTTAAAAGC |
| C713A | forward | GGAATGGGCTAAGTACGAACG |
|  | reverse | CGTACTTAGCCCATTCCGACC |
| C750A | forward | ACGCTTTGCCAAACTGCGC |
|  | reverse | CAGTTTGGCAAAGCGTTGAGG |

**Table S2. Gene constructs for expression of WT and mutants of TLR2_TIR_.** Mutations are indicated in bold.

| gene | sequence |
| --- | --- |
| WT | atgcatcaccatcaccatcacggttctggttctggtctggttccgcgtggatccagccgtaagatttgttacgacgcatttgttagctactcggaacgcgatgcctactgggttgaaaatctgatggttcaggaactggagaacttcaatccgccttttaaactgtgcctgcataaacgcgatttcatcccaggcaagtggatcattgacaacattattgacagcatcgagaagtcgcataagaccgtgttcgtcctgtcggagaactttgttaagtcggaatggtgtaagtacgaactggacttcagccacttccgcctgtttgacgaaaacaatgatgctgcgattctgatcctcctggaaccaatcgaaaaaaaagctattcctcaacgcttttgcaaactgcgcaaaatcatgaacactaaaacctacctggagtggcctatggatgaggcgcaacgcgaaggtttttgggtaaatctgcgtgcagctattaaatcgt |
| 4xCYS | atgcatcaccatcaccatcacggttctggttctggtctggttccgcgtggatccagccgtaagatt**gct**tacgacgcatttgttagctactcggaacgcgatgcctactgggttgaaaatctgatggttcaggaactggagaacttcaatccgccttttaaactg**gcc**ctgcataaacgcgatttcatcccaggcaagtggatcattgacaacattqu waattgacagcatcgagaagtcgcataagaccgtgttcgtcctgtcggagaactttgttaagtcggaatgg**gct**aagtacgaactggacttcagccacttccgcctgtttgacgaaaacaatgatgctgcgattctgatcctcctggaaccaatcgaaaaaaaagctattcctcaacgcttt**gcc**aaactgcgcaaaatcatgaacactaaaacctacctggagtggcctatggatgaggcgcaacgcgaaggtttttgggtaaatctgcgtgcagctattaaatcgt |
| C640A | atgcatcaccatcaccatcacggttctggttctggtctggttccgcgtggatccagccgtaagatt**gct**tacgacgcatttgttagctactcggaacgcgatgcctactgggttgaaaatctgatggttcaggaactggagaacttcaatccgccttttaaactgtgcctgcataaacgcgatttcatcccaggcaagtggatcattgacaacattattgacagcatcgagaagtcgcataagaccgtgttcgtcctgtcggagaactttgttaagtcggaatggtgtaagtacgaactggacttcagccacttccgcctgtttgacgaaaacaatgatgctgcgattctgatcctcctggaaccaatcgaaaaaaaagctattcctcaacgcttttgcaaactgcgcaaaatcatgaacactaaaacctacctggagtggcctatggatgaggcgcaacgcgaaggtttttgggtaaatctgcgtgcagctattaaatcgt |
| C673A | atgcatcaccatcaccatcacggttctggttctggtctggttccgcgtggatccagccgtaagatttgttacgacgcatttgttagctactcggaacgcgatgcctactgggttgaaaatctgatggttcaggaactggagaacttcaatccgccttttaaactg**gcc**ctgcataaacgcgatttcatcccaggcaagtggatcattgacaacattattgacagcatcgagaagtcgcataagaccgtgttcgtcctgtcggagaactttgttaagtcggaatggtgtaagtacgaactggacttcagccacttccgcctgtttgacgaaaacaatgatgctgcgattctgatcctcctggaaccaatcgaaaaaaaagctattcctcaacgcttttgcaaactgcgcaaaatcatgaacactaaaacctacctggagtggcctatggatgaggcgcaacgcgaaggtttttgggtaaatctgcgtgcagctattaaatcgt |
| C713A | atgcatcaccatcaccatcacggttctggttctggtctggttccgcgtggatccagccgtaagatttgttacgacgcatttgttagctactcggaacgcgatgcctactgggttgaaaatctgatggttcaggaactggagaacttcaatccgccttttaaactgtgcctgcataaacgcgatttcatcccaggcaagtggatcattgacaacattattgacagcatcgagaagtcgcataagaccgtgttcgtcctgtcggagaactttgttaagtcggaatgg**gct**aagtacgaactggacttcagccacttccgcctgtttgacgaaaacaatgatgctgcgattctgatcctcctggaaccaatcgaaaaaaaagctattcctcaacgcttttgcaaactgcgcaaaatcatgaacactaaaacctacctggagtggcctatggatgaggcgcaacgcgaaggtttttgggtaaatctgcgtgcagctattaaatcgt |
| C750A | atgcatcaccatcaccatcacggttctggttctggtctggttccgcgtggatccagccgtaagatttgttacgacgcatttgttagctactcggaacgcgatgcctactgggttgaaaatctgatggttcaggaactggagaacttcaatccgccttttaaactgtgcctgcataaacgcgatttcatcccaggcaagtggatcattgacaacattattgacagcatcgagaagtcgcataagaccgtgttcgtcctgtcggagaactttgttaagtcggaatggtgtaagtacgaactggacttcagccacttccgcctgtttgacgaaaacaatgatgctgcgattctgatcctcctggaaccaatcgaaaaaaaagctattcctcaacgcttt**gcc**aaactgcgcaaaatcatgaacactaaaacctacctggagtggcctatggatgaggcgcaacgcgaaggtttttgggtaaatctgcgtgcagctattaaatcgt |
| C640/673A | atgcatcaccatcaccatcacggttctggttctggtctggttccgcgtggatccagccgtaagatt**gct**tacgacgcatttgttagctactcggaacgcgatgcctactgggttgaaaatctgatggttcaggaactggagaacttcaatccgccttttaaactg**gcc**ctgcataaacgcgatttcatcccaggcaagtggatcattgacaacattattgacagcatcgagaagtcgcataagaccgtgttcgtcctgtcggagaactttgttaagtcggaatggtgtaagtacgaactggacttcagccacttccgcctgtttgacgaaaacaatgatgctgcgattctgatcctcctggaaccaatcgaaaaaaaagctattcctcaacgcttttgcaaactgcgcaaaatcatgaacactaaaacctacctggagtggcctatggatgaggcgcaacgcgaaggtttttgggtaaatctgcgtgcagctattaaatcgt |
| C640/750A | atgcatcaccatcaccatcacggttctggttctggtctggttccgcgtggatccagccgtaagatt**gct**tacgacgcatttgttagctactcggaacgcgatgcctactgggttgaaaatctgatggttcaggaactggagaacttcaatccgccttttaaactgtgcctgcataaacgcgatttcatcccaggcaagtggatcattgacaacattattgacagcatcgagaagtcgcataagaccgtgttcgtcctgtcggagaactttgttaagtcggaatggtgtaagtacgaactggacttcagccacttccgcctgtttgacgaaaacaatgatgctgcgattctgatcctcctggaaccaatcgaaaaaaaagctattcctcaacgcttt**gcc**aaactgcgcaaaatcatgaacactaaaacctacctggagtggcctatggatgaggcgcaacgcgaaggtttttgggtaaatctgcgtgcagctattaaatcgt |
| C673/713A | atgcatcaccatcaccatcacggttctggttctggtctggttccgcgtggatccagccgtaagatttgttacgacgcatttgttagctactcggaacgcgatgcctactgggttgaaaatctgatggttcaggaactggagaacttcaatccgccttttaaactg**gcc**ctgcataaacgcgatttcatcccaggcaagtggatcattgacaacattattgacagcatcgagaagtcgcataagaccgtgttcgtcctgtcggagaactttgttaagtcggaatgg**gct**aagtacgaactggacttcagccacttccgcctgtttgacgaaaacaatgatgctgcgattctgatcctcctggaaccaatcgaaaaaaaagctattcctcaacgcttttgcaaactgcgcaaaatcatgaacactaaaacctacctggagtggcctatggatgaggcgcaacgcgaaggtttttgggtaaatctgcgtgcagctattaaatcgt |
| C713/750A | atgcatcaccatcaccatcacggttctggttctggtctggttccgcgtggatccagccgtaagatttgttacgacgcatttgttagctactcggaacgcgatgcctactgggttgaaaatctgatggttcaggaactggagaacttcaatccgccttttaaactgtgcctgcataaacgcgatttcatcccaggcaagtggatcattgacaacattattgacagcatcgagaagtcgcataagaccgtgttcgtcctgtcggagaactttgttaagtcggaatgg**gct**aagtacgaactggacttcagccacttccgcctgtttgacgaaaacaatgatgctgcgattctgatcctcctggaaccaatcgaaaaaaaagctattcctcaacgcttt**gcc**aaactgcgcaaaatcatgaacactaaaacctacctggagtggcctatggatgaggcgcaacgcgaaggtttttgggtaaatctgcgtgcagctattaaatcgt |

**Table S3.** **Amino acid sequence of WT and mutants of TLR2_TIR_.** N-terminal tags are shown in bold. Mutations highlighted by underlining, bold.

| protein | aa sequence of H6-TLR2_TIR_ hybrid |
| --- | --- |
| WT | **MHHHHHHGSGSGLVPRGS**SRKICYDAFVSYSERDAYWVENLMVQELENFNPPFKLCLHKRDFIPGKWIIDNIIDSIEKSHKTVFVLSENFVKSEWCKYELDFSHFRLFDENNDAAILILLEPIEKKAIPQRFCKLRKIMNTKTYLEWPMDEAQREGFWVNLRAAIKS |
| 4xCYS | **MHHHHHHGSGSGLVPRGS**SRKI**A**YDAFVSYSERDAYWVENLMVQELENFNPPFKL**A**LHKRDFIPGKWIIDNIIDSIEKSHKTVFVLSENFVKSEW**A**KYELDFSHFRLFDENNDAAILILLEPIEKKAIPQRF**A**KLRKIMNTKTYLEWPMDEAQREGFWVNLRAAIKS |
| C640A | **MHHHHHHGSGSGLVPRGS**SRKI**A**YDAFVSYSERDAYWVENLMVQELENFNPPFKLCLHKRDFIPGKWIIDNIIDSIEKSHKTVFVLSENFVKSEWCKYELDFSHFRLFDENNDAAILILLEPIEKKAIPQRFCKLRKIMNTKTYLEWPMDEAQREGFWVNLRAAIKS |
| C673A | **MHHHHHHGSGSGLVPRGS**SRKICYDAFVSYSERDAYWVENLMVQELENFNPPFKL**A**LHKRDFIPGKWIIDNIIDSIEKSHKTVFVLSENFVKSEWCKYELDFSHFRLFDENNDAAILILLEPIEKKAIPQRFCKLRKIMNTKTYLEWPMDEAQREGFWVNLRAAIKS |
| C713A | **MHHHHHHGSGSGLVPRGS**SRKICYDAFVSYSERDAYWVENLMVQELENFNPPFKLCLHKRDFIPGKWIIDNIIDSIEKSHKTVFVLSENFVKSEW**A**KYELDFSHFRLFDENNDAAILILLEPIEKKAIPQRFCKLRKIMNTKTYLEWPMDEAQREGFWVNLRAAIKS |
| C750A | **MHHHHHHGSGSGLVPRGS**SRKICYDAFVSYSERDAYWVENLMVQELENFNPPFKLCLHKRDFIPGKWIIDNIIDSIEKSHKTVFVLSENFVKSEWCKYELDFSHFRLFDENNDAAILILLEPIEKKAIPQRF**A**KLRKIMNTKTYLEWPMDEAQREGFWVNLRAAIKS |
| C640/673A | **MHHHHHHGSGSGLVPRGS**SRKI**A**YDAFVSYSERDAYWVENLMVQELENFNPPFKL**A**LHKRDFIPGKWIIDNIIDSIEKSHKTVFVLSENFVKSEWCKYELDFSHFRLFDENNDAAILILLEPIEKKAIPQRFCKLRKIMNTKTYLEWPMDEAQREGFWVNLRAAIKS |
| C640/750A | **MHHHHHHGSGSGLVPRGS**SRKI**A**YDAFVSYSERDAYWVENLMVQELENFNPPFKLCLHKRDFIPGKWIIDNIIDSIEKSHKTVFVLSENFVKSEWCKYELDFSHFRLFDENNDAAILILLEPIEKKAIPQRF**A**KLRKIMNTKTYLEWPMDEAQREGFWVNLRAAIKS |
| C673/713A | **MHHHHHHGSGSGLVPRGS**SRKICYDAFVSYSERDAYWVENLMVQELENFNPPFKL**A**LHKRDFIPGKWIIDNIIDSIEKSHKTVFVLSENFVKSEW**A**KYELDFSHFRLFDENNDAAILILLEPIEKKAIPQRFCKLRKIMNTKTYLEWPMDEAQREGFWVNLRAAIKS |
| C713/750A | **MHHHHHHGSGSGLVPRGS**SRKICYDAFVSYSERDAYWVENLMVQELENFNPPFKLCLHKRDFIPGKWIIDNIIDSIEKSHKTVFVLSENFVKSEW**A**KYELDFSHFRLFDENNDAAILILLEPIEKKAIPQRF**A**KLRKIMNTKTYLEWPMDEAQREGFWVNLRAAIKS |


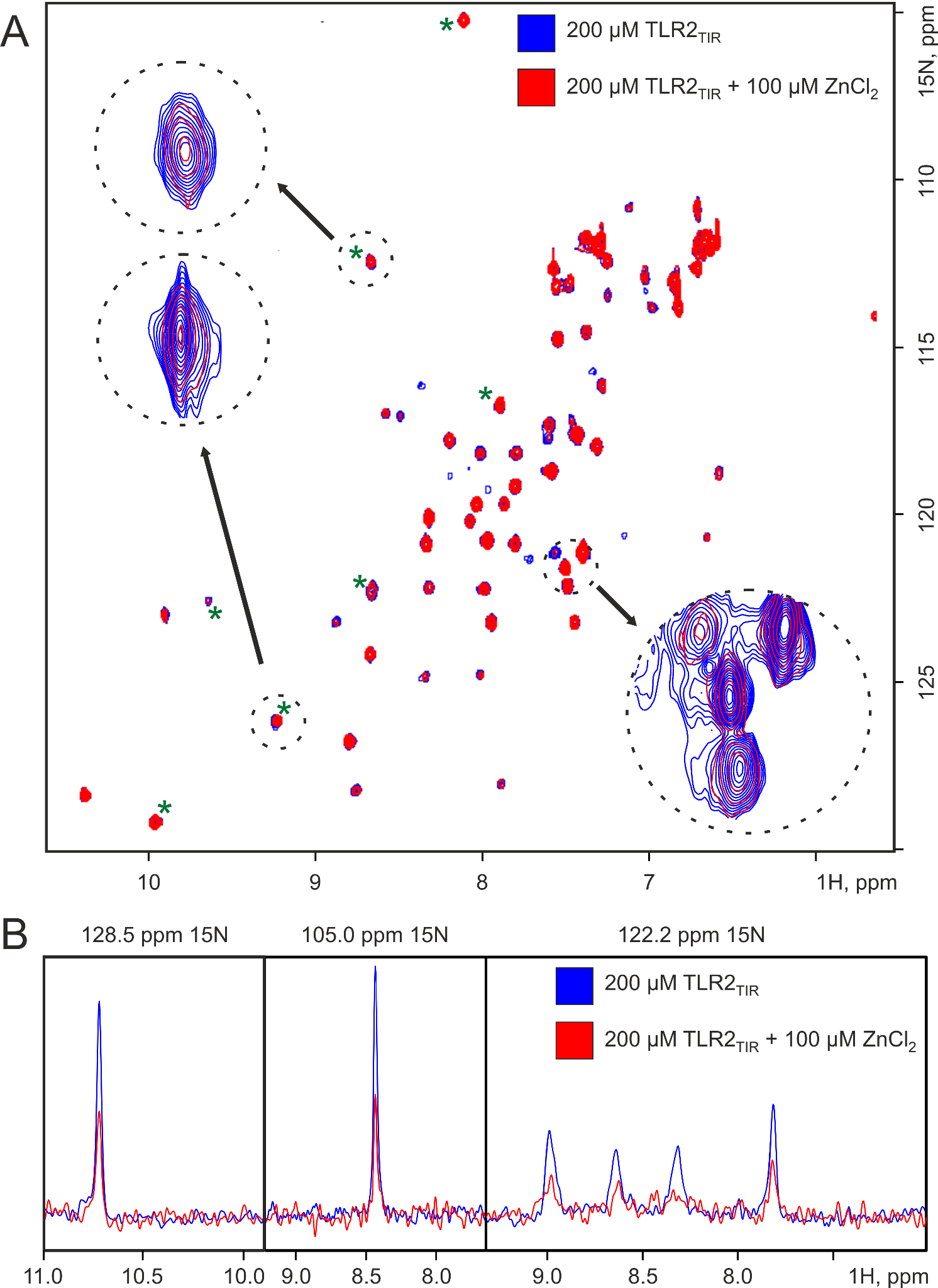


**Fig. S1. The effect of adding zinc to TLR2_TIR_. A -** Superposition of ^1^H,^15^N-HSQC spectra of 200 µM WT TLR2_TIR_ before and after addition of 100 µM ZnCl**_2_**. The wild type TLR2_TIR_ without zinc ions is in blue, and after addition of ZnCl_2_ is in red. Green stars indicate signals used to calculate the concentration of free TLR2_TIR_. To indicate a decrease in signal intensity, enlarged regions of the spectrum are shown in dotted circles. **B -** One-dimensional slices of superposition of ^1^H,^15^N-HSQC spectra of TLR2_TIR_ before and after addition of 100 µM ZnCl**_2_**. The slices were created using spectra from panel A. The wild type TLR2_TIR_ without zinc ions is in blue, and after addition of ZnCl_2_ is in red.


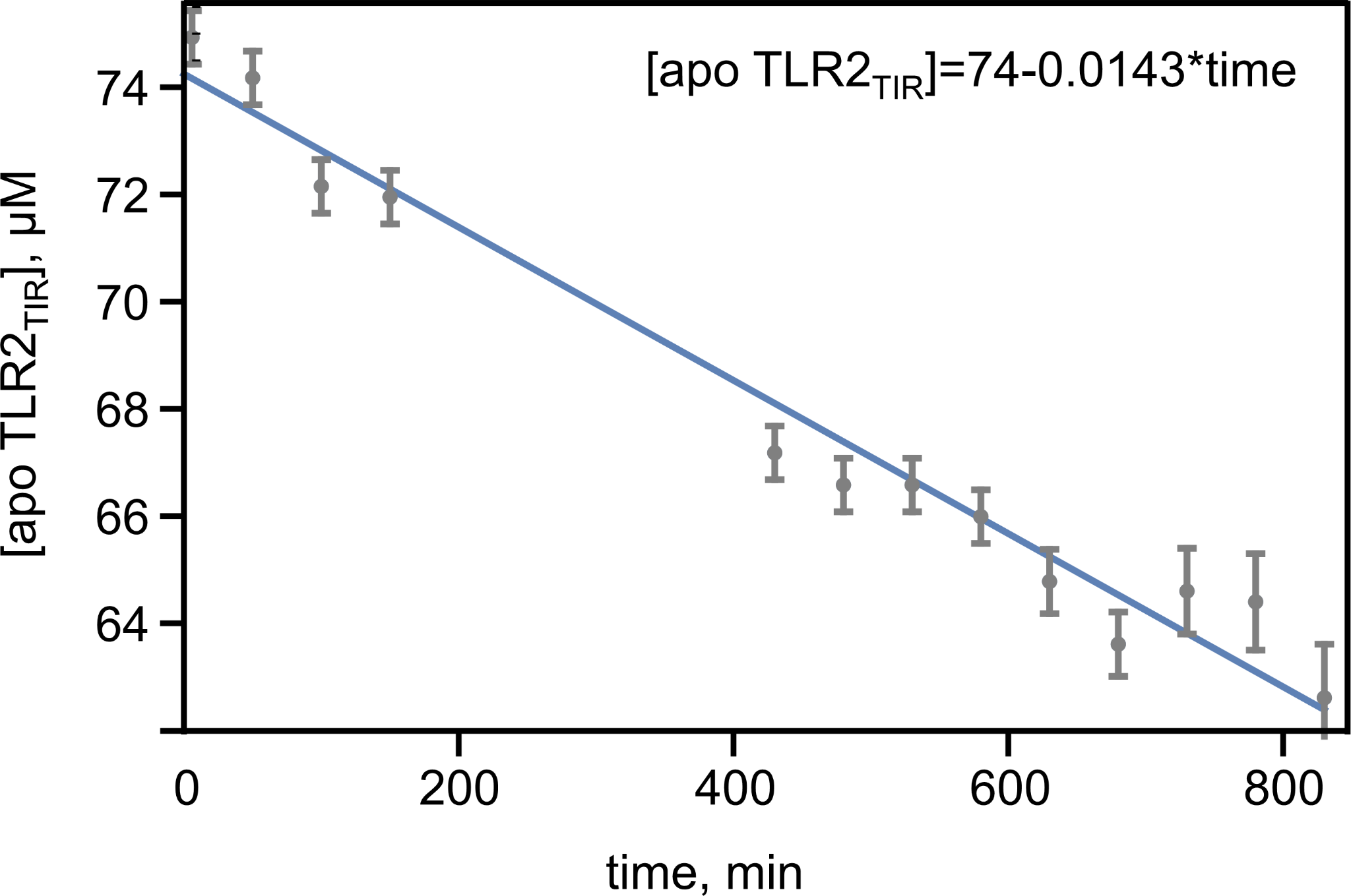


**Fig.S2. Determination of the average rate of TLR2_TIR_ oligomerization.** The experimental points are shown in gray, taking into account the signal-to-noise error and averaging over the analyzed signals. The approximation of the protein concentration decline is shown in gray-blue. The value of the rate of the protein concentration decline was determined from the slope of the approximation line. The measurement error was determined from the average value of the analyzed peaks and the signal-to-noise ratio.


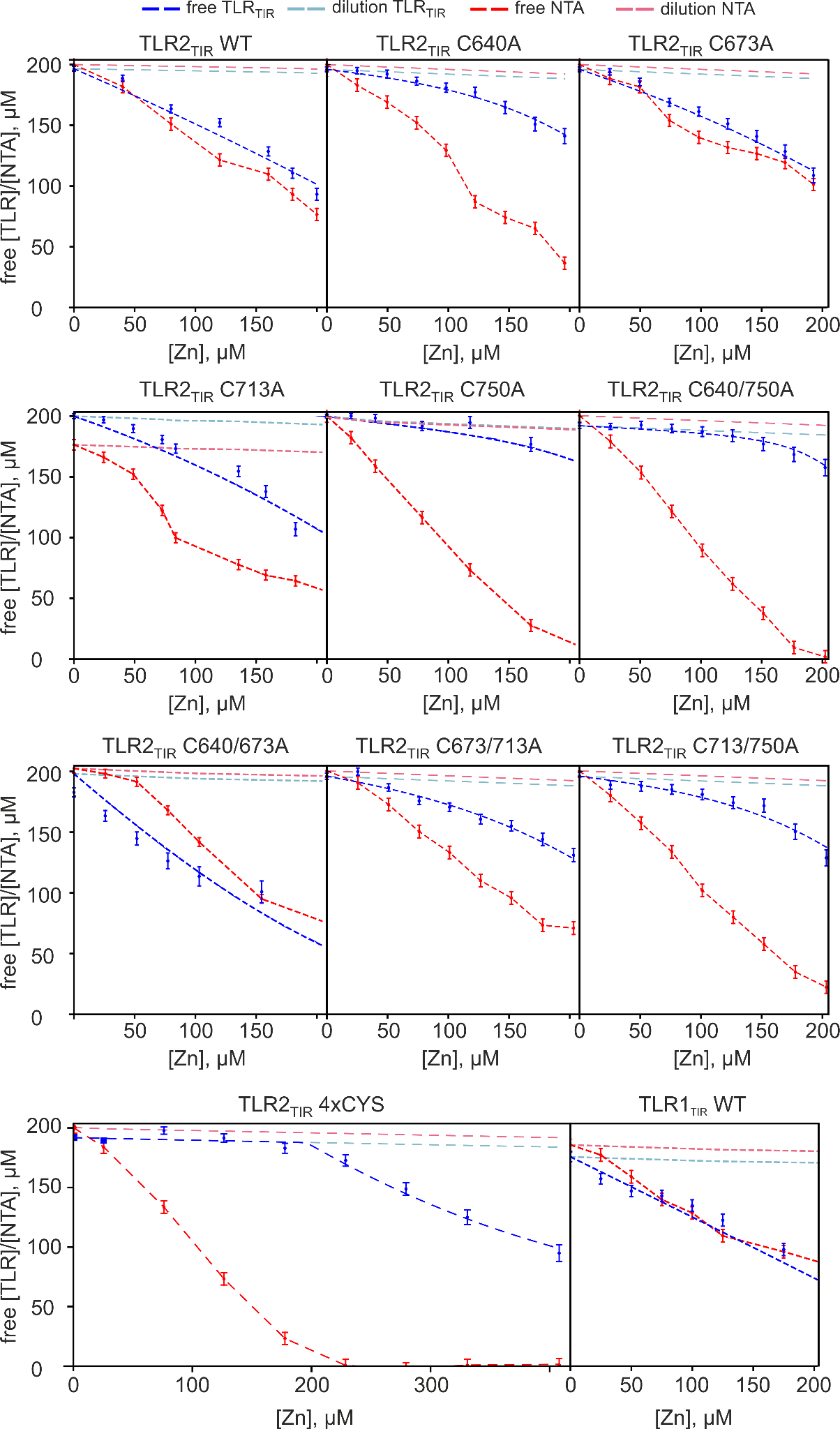


**Fig. S3. Titration of WT TLR1_TIR_, WT TLR2_TIR_ and its mutants with Zn in the presence of NTA.** The concentrations of apo TLR2_TIR_ and unbound NTA are plotted as a function of Zn concentration by blue and red dots, respectively. The theoretical dependencies corresponding to the respective K_d_ are represented by a dashed blue line for TLR2_TIR_. The concentration of free protein was obtained from the analysis of peak intensities in ^1^H,^15^N-HSQC NMR spectra. The measurement error was determined from the average value of the analyzed peaks and the signal-to-noise ratio.


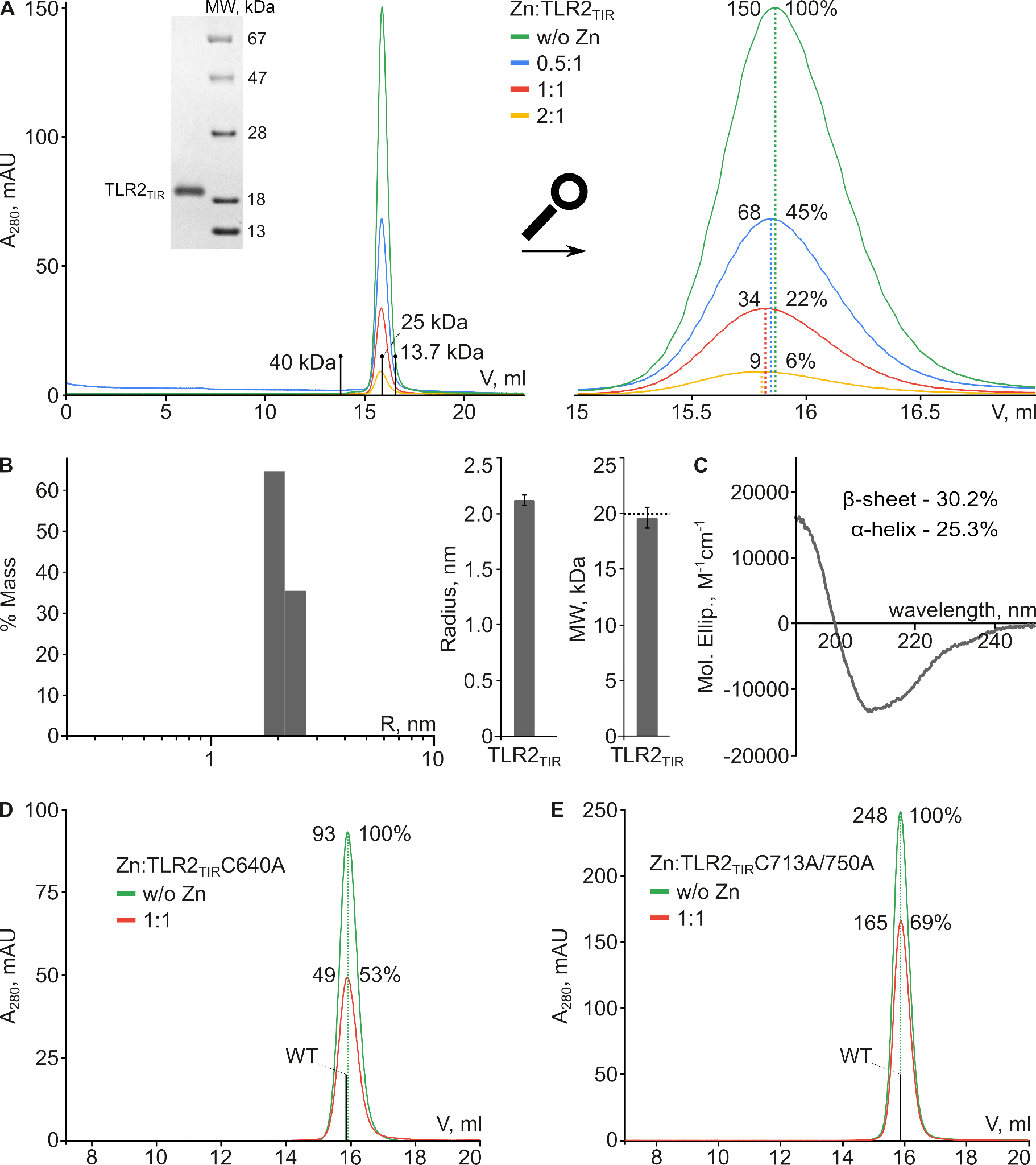


**Fig. S4. Analysis of TLR2_TIR_ samples.** **A.** SEC profiles of wild-type TLR2_TIR_ with and without zinc. SDS-page gel shows the sample purity. Protein concentration was 40 µM. **B.** Distribution of particle sizes (left), the measured average radius (middle) and the molecular weight (right) of the elution peak from the SEC, obtained by the DLS. The dotted line indicates the calculated molecular weight of TLR2_TIR_. **C.** CD-spectra analysis of wild-type TLR2_TIR_. **D-E.** SEC profiles of TLR2_TIR_ С640A and C713A/C750A with and without zinc.


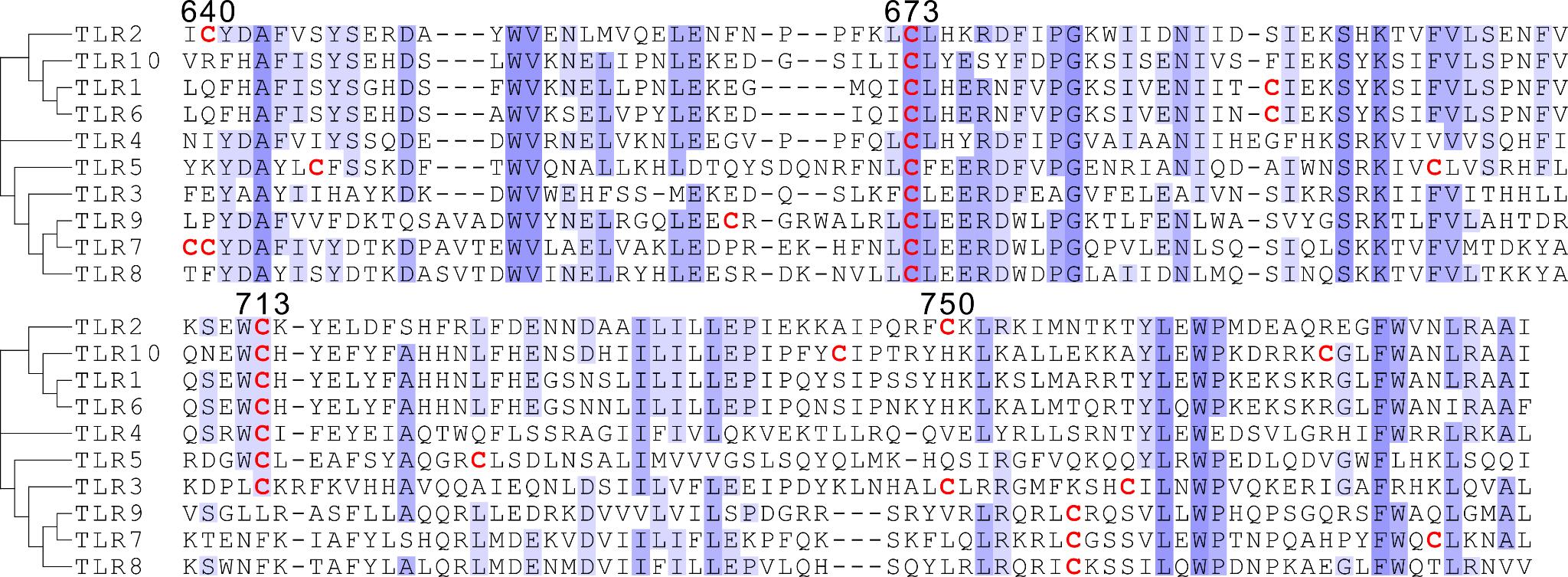


**Fig. S5.** **Alignment of protein sequences of all the human TLR TIR.** The cysteine residues are marked in bold red, conservative residues are highlighted in blue. The gene tree is shown on the left. The UniProt Align (https://www.uniprot.org/align) tool was used for protein alignments.


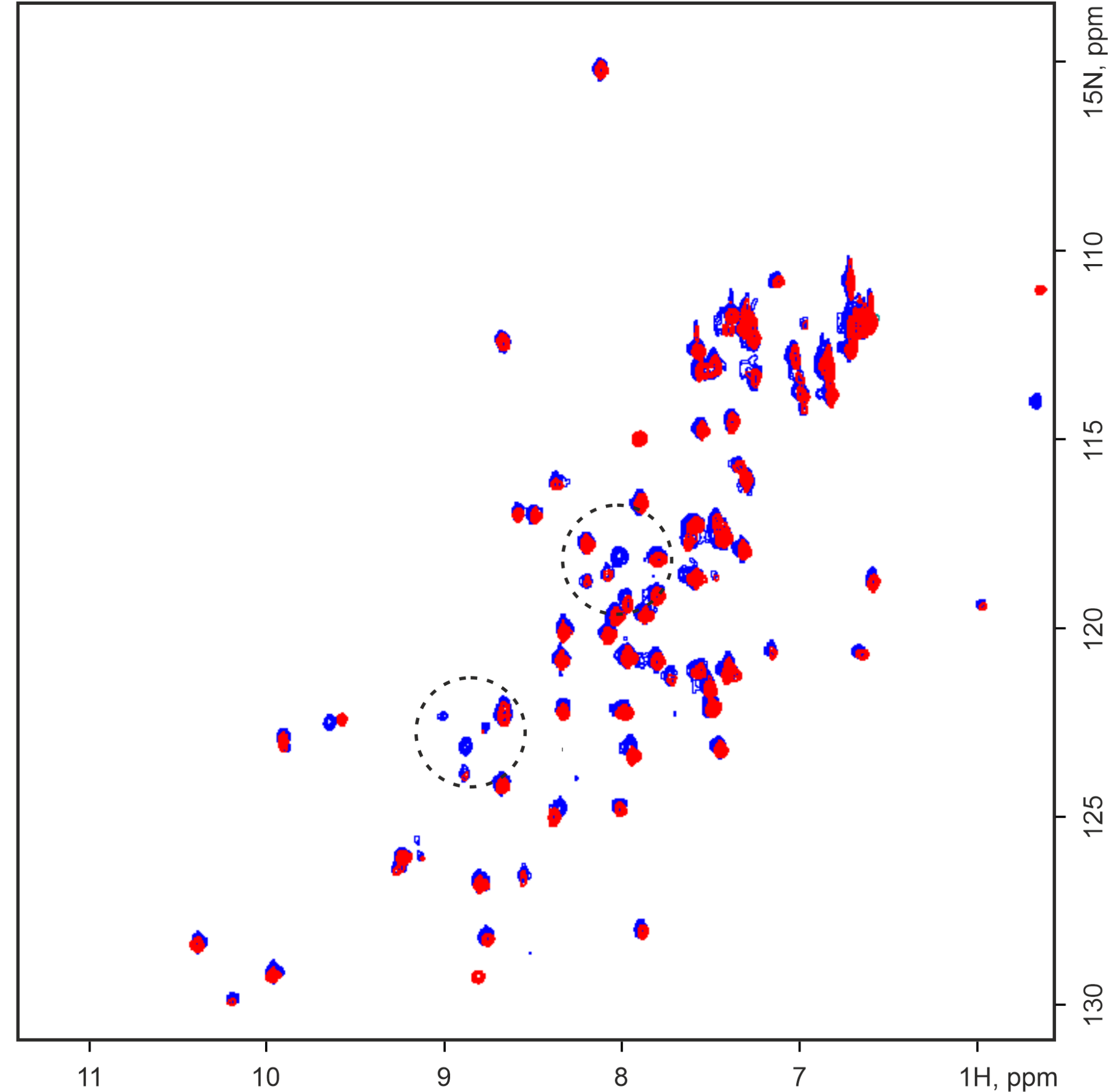


**Fig. S6. Superposition of ^1^H,^15^N-HSQC spectra of WT TLR2_TIR_ and TLR2_TIR_ C640A**. The wild type TLR2_TIR_ is in blue, and the mutant form is in red. The black dotted line outlines the areas with changes in the NMR spectra.


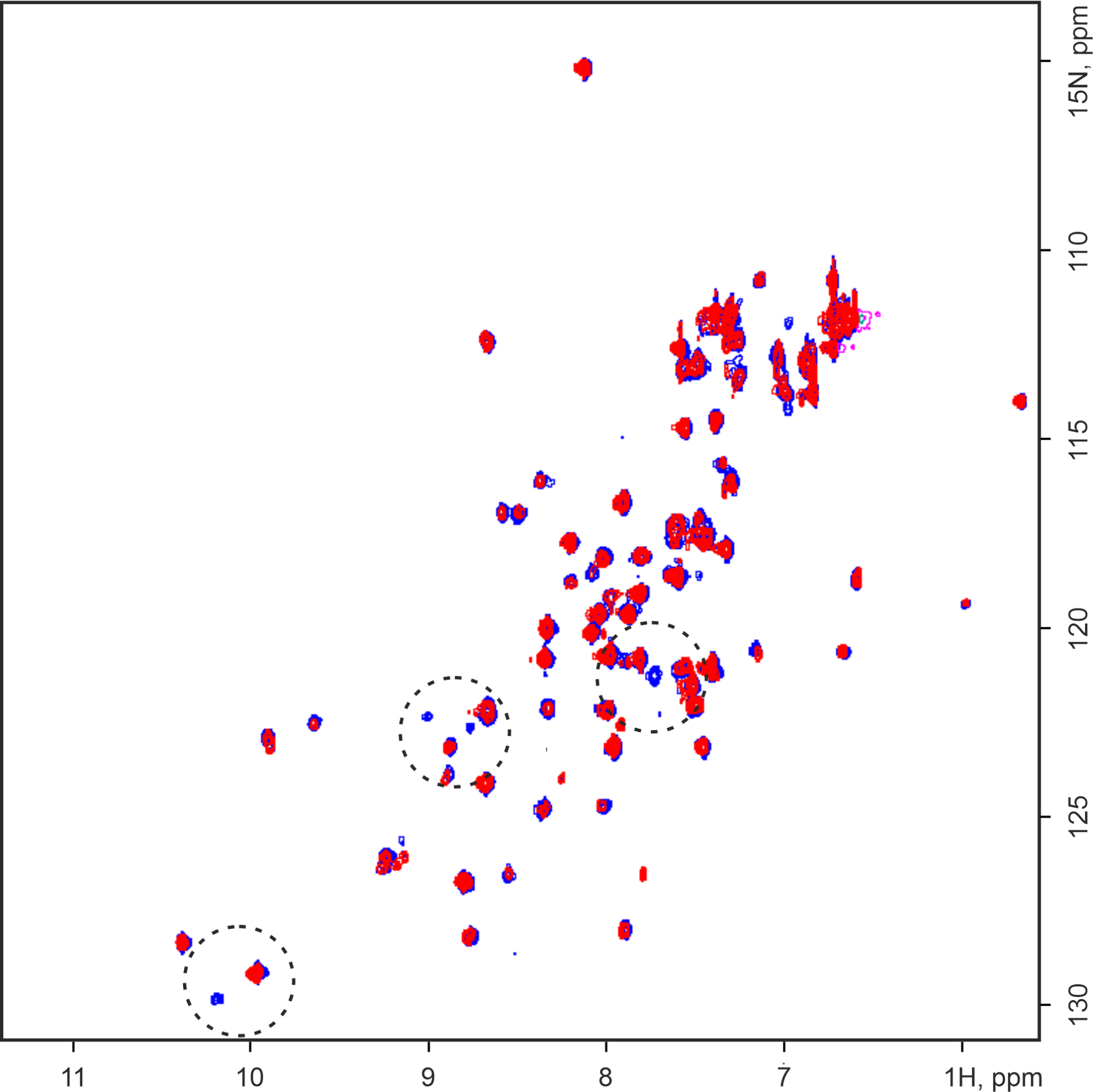


**Fig. S7. Superposition of ^1^H,^15^N-HSQC spectra of WT TLR2_TIR_ and TLR2_TIR_ C673A**. The wild type TLR2_TIR_ is in blue, and the mutant form is in red. The black dotted line outlines the areas with changes in the NMR spectra.


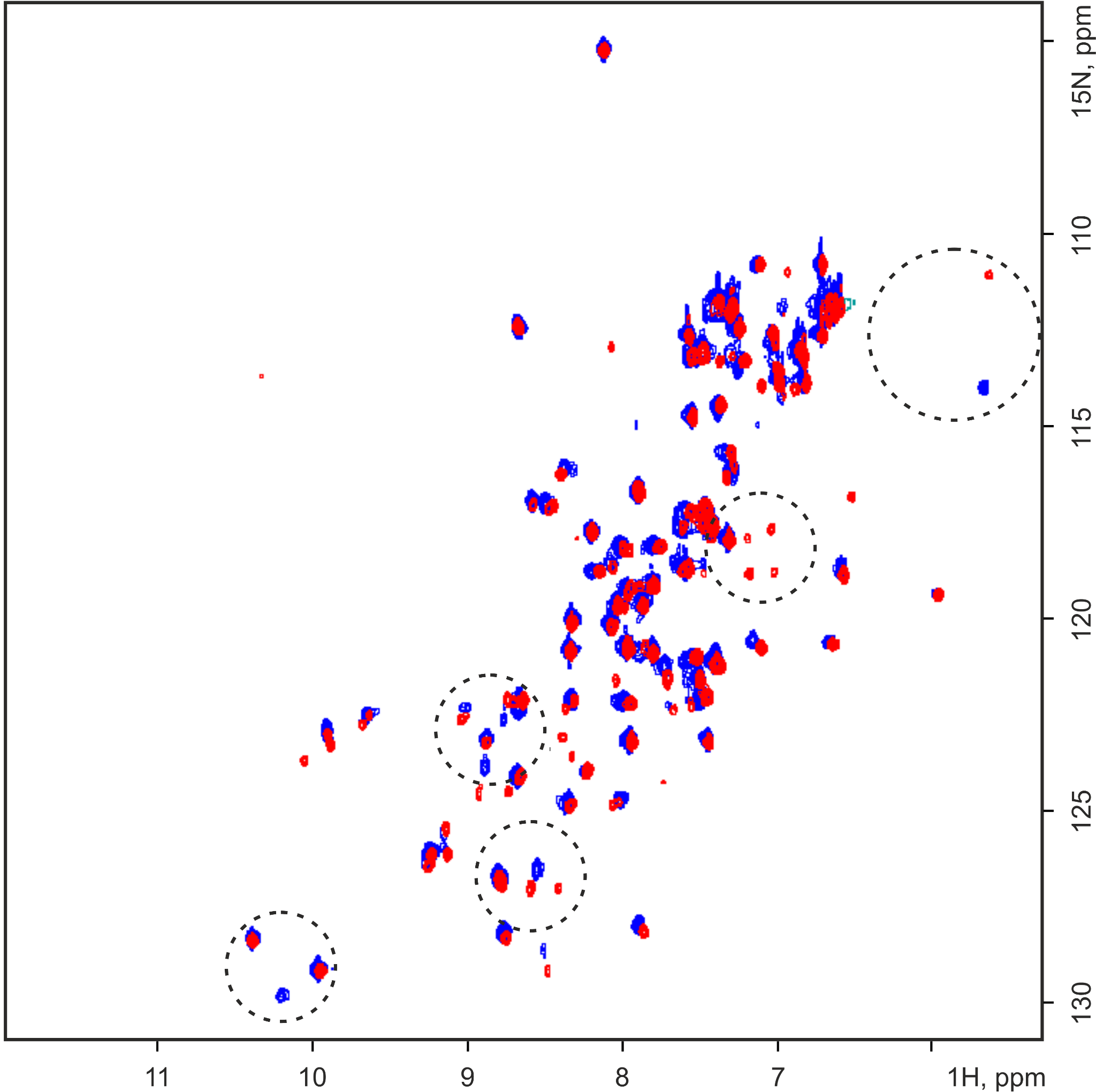


**Fig. S8. Superposition of ^1^H,^15^N-HSQC spectra of WT TLR2_TIR_ and TLR2_TIR_ C713A**. The wild type TLR2_TIR_ is in blue, and the mutant form is in red. The black dotted line outlines the areas with changes in the NMR spectra.


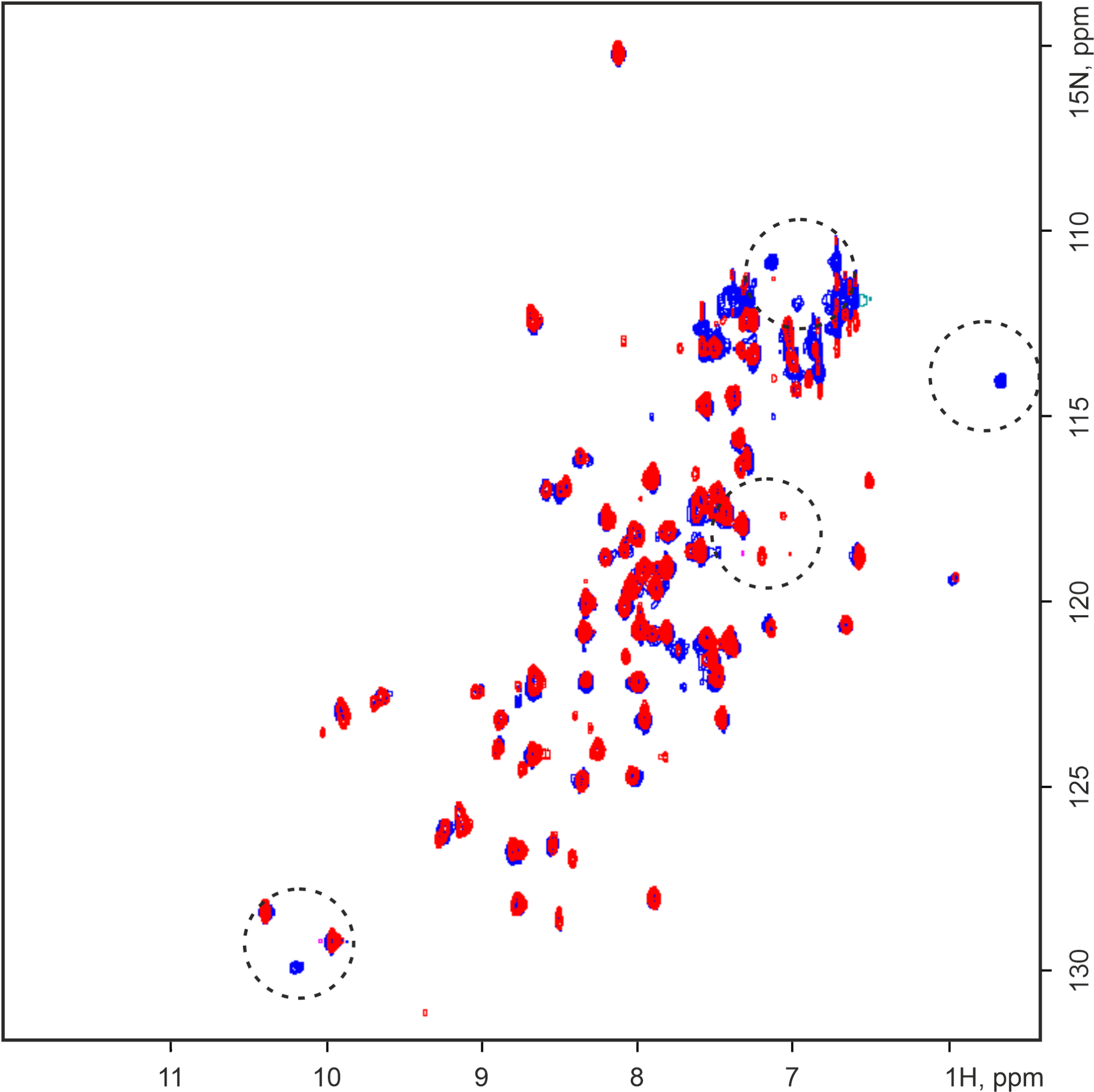


**Fig. S9. Superposition of ^1^H,^15^N-HSQC spectra of WT TLR2_TIR_ and TLR2_TIR_ C750A**. The wild type TLR2_TIR_ is in blue, and the mutant form is in red. The black dotted line outlines the areas with changes in the NMR spectra.


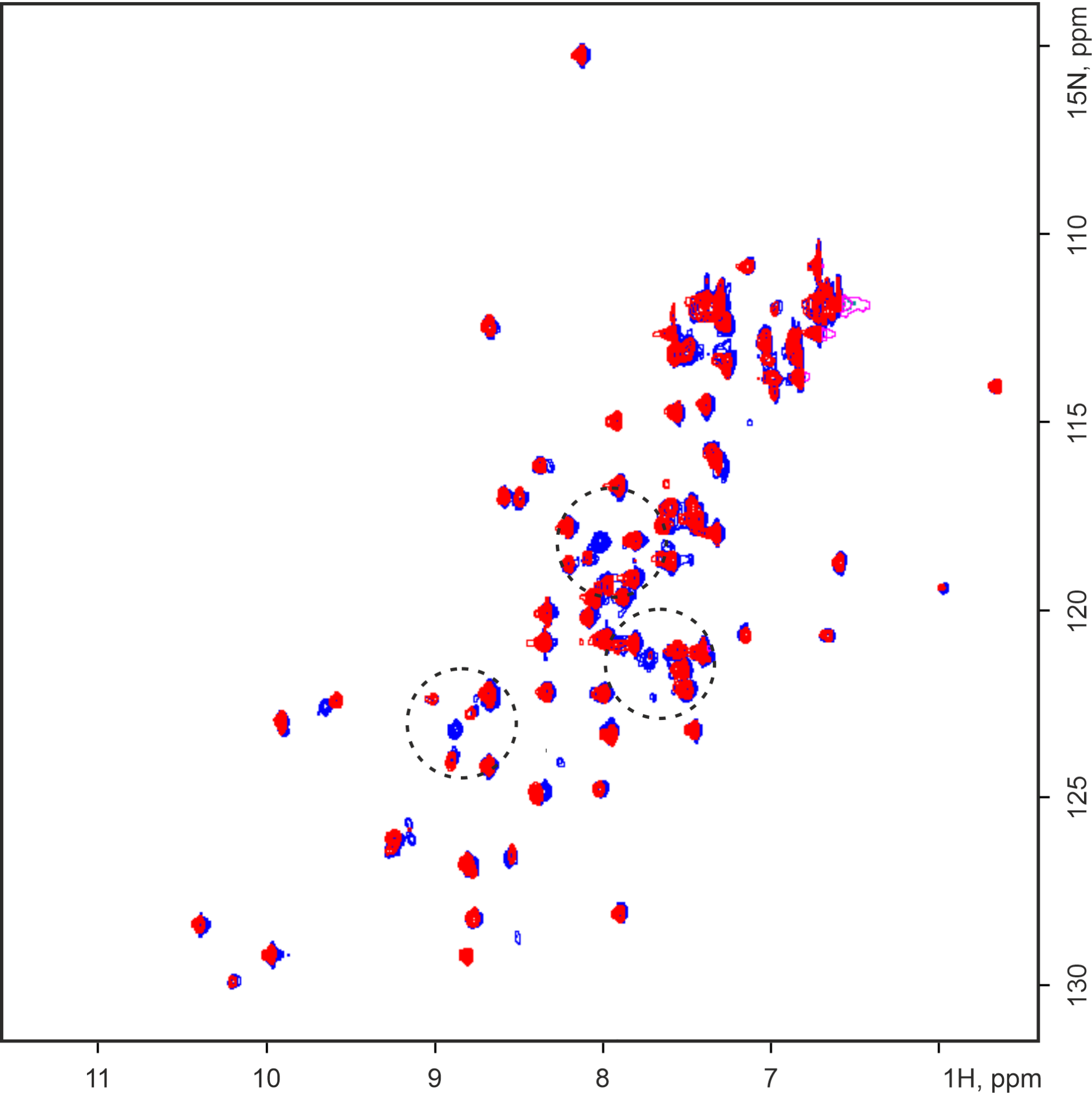


**Fig. S10. Superposition of ^1^H,^15^N-HSQC spectra of WT TLR2_TIR_ and TLR2_TIR_ C640/750A**. The wild type TLR2_TIR_ is in blue, and the mutant form is in red. The black dotted line outlines the areas with changes in the NMR spectra.


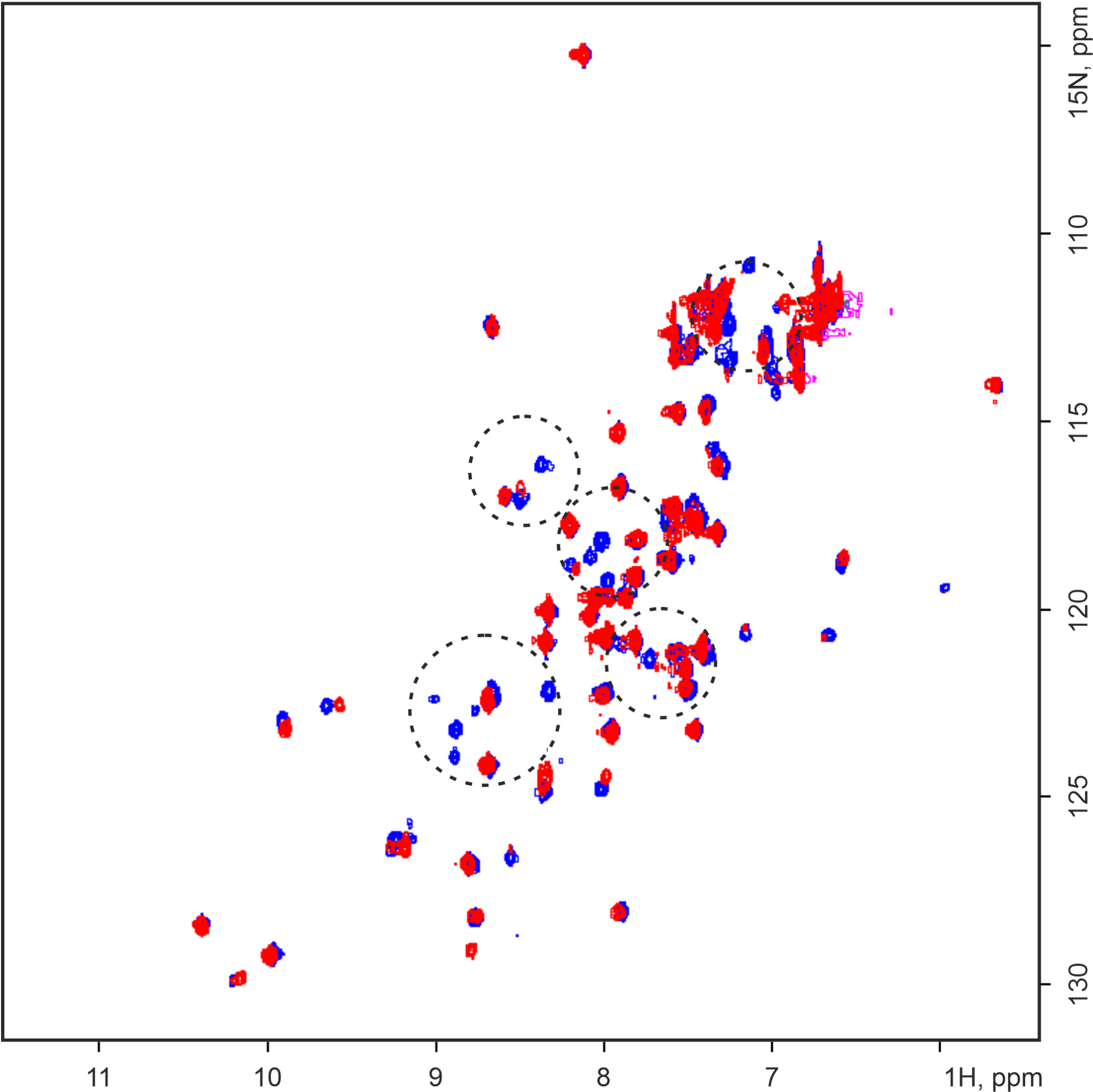


**Fig. S11. Superposition of ^1^H,^15^N-HSQC spectra of WT TLR2_TIR_ and TLR2_TIR_ C640/673A**. The wild type TLR2_TIR_ is in blue, and the mutant form is in red. The black dotted line outlines the areas with changes in the NMR spectra.


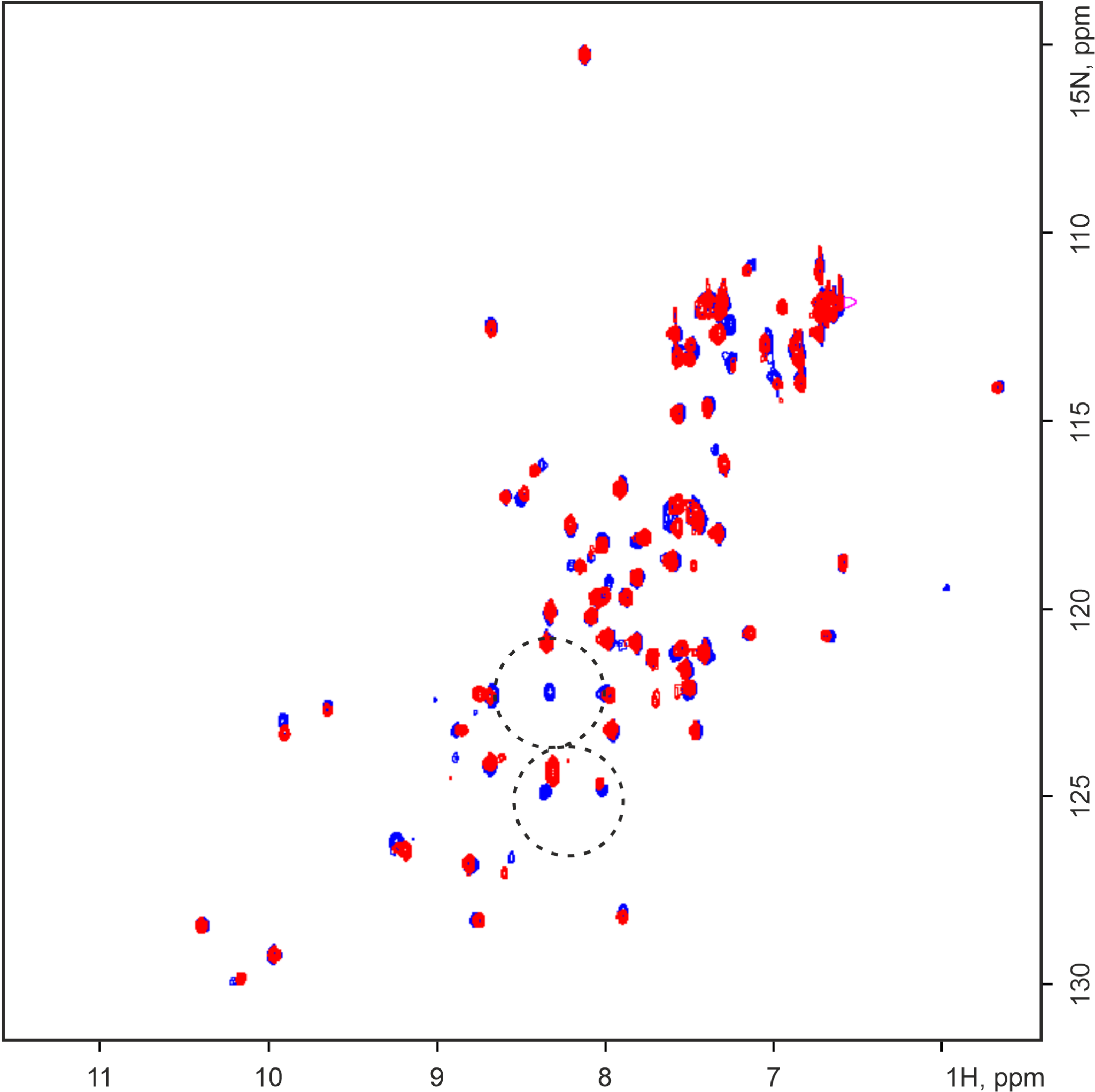


**Fig. S12. Superposition of ^1^H,^15^N-HSQC spectra of WT TLR2_TIR_ and TLR2_TIR_ C673/713A**. The wild type TLR2_TIR_ is in blue, and the mutant form is in red. The black dotted line outlines the areas with changes in the NMR spectra.


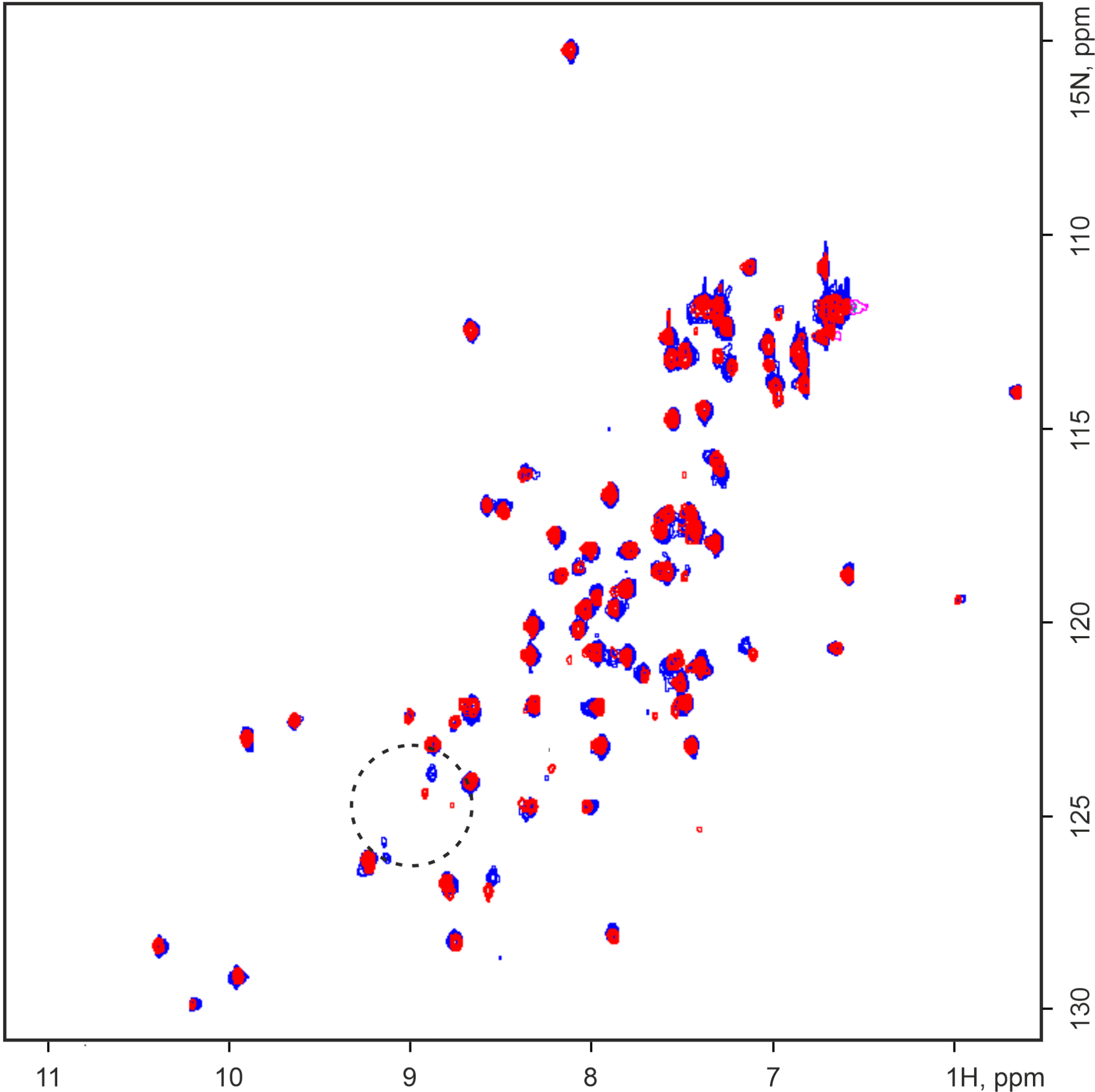


**Fig. S13. Superposition of ^1^H,^15^N-HSQC spectra of WT TLR2_TIR_ and TLR2_TIR_ C713/750A**. The wild type TLR2_TIR_ is in blue, and the mutant form is in red. The black dotted line outlines the areas with changes in the NMR spectra.


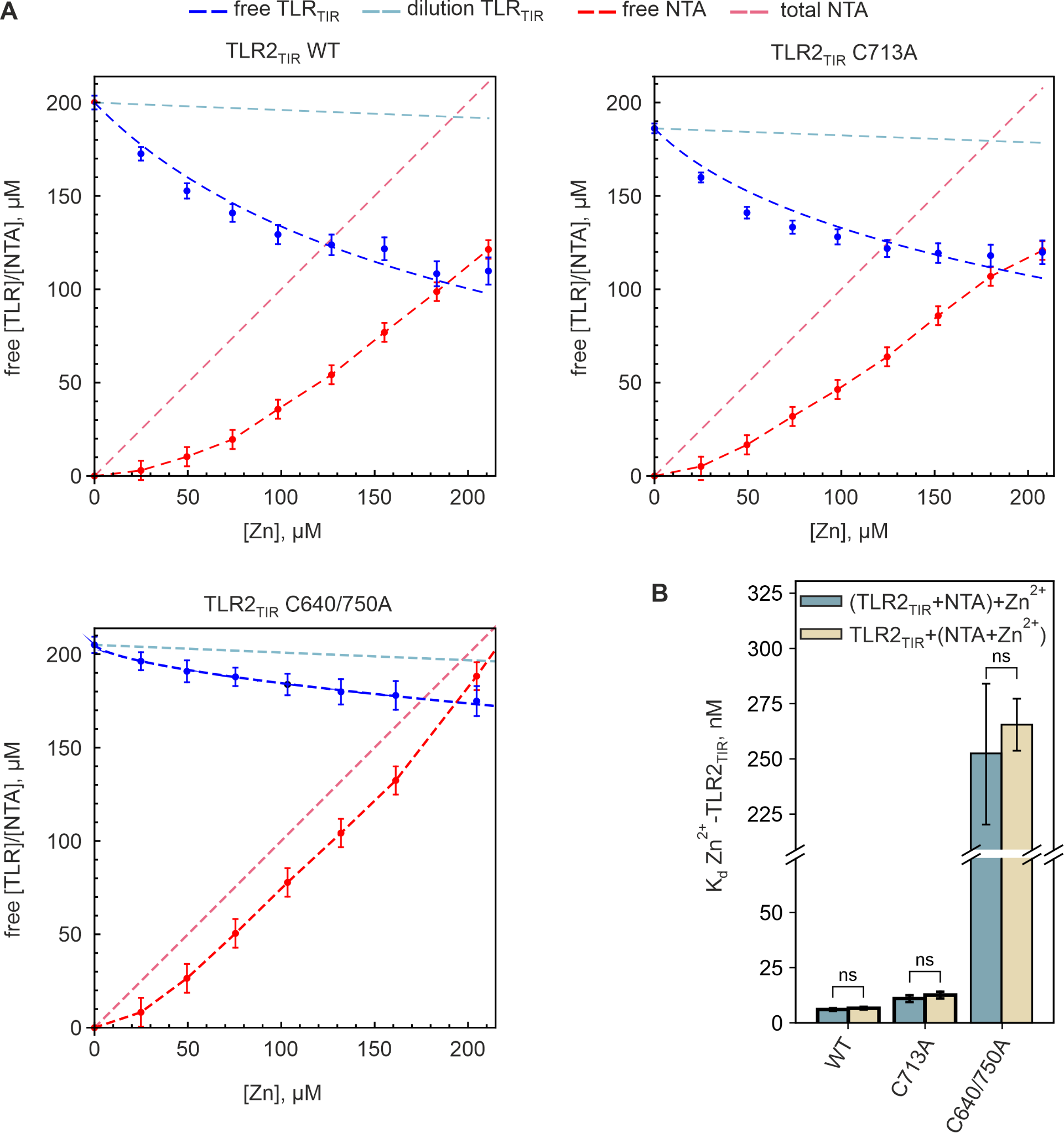


**Fig. S14. The analysis of signal intensity changes in the competitive binding experiments for WT, C713 and C640/750A mutants.** Each protein was titrated by a mixture of NTA:zinc (1:1) and the concentration of free TLR2_TIR_ was obtained from the analysis of peak intensities in ^1^H,^15^N-HSQC NMR spectra. **A**. Titration curves of TLR2_TIR_ WT, C713 and C640/750A. The concentrations of apo TLR2_TIR_ and unbound NTA are plotted as a function of zinc concentration by blue and red dots, respectively. The theoretical dependencies corresponding to Kd are shown as a dashed blue line for TLR2_TIR_. The measurement error was determined from the average value of the analyzed peaks and the signal-to-noise ratio. **B.** Сomparison of the K_d_ values obtained by two titration methods: TLR2_TIR_:NTA (1:1) upon addition of Zn (grey blue) and TLR2_TIR_ upon addition of Zn:NTA (1:1) (sand). Statistical analysis was carried out based on the calculation of t-statistics, and “ns” denotes that changes are not significant. Error bars indicate the standard deviations (measurement error).


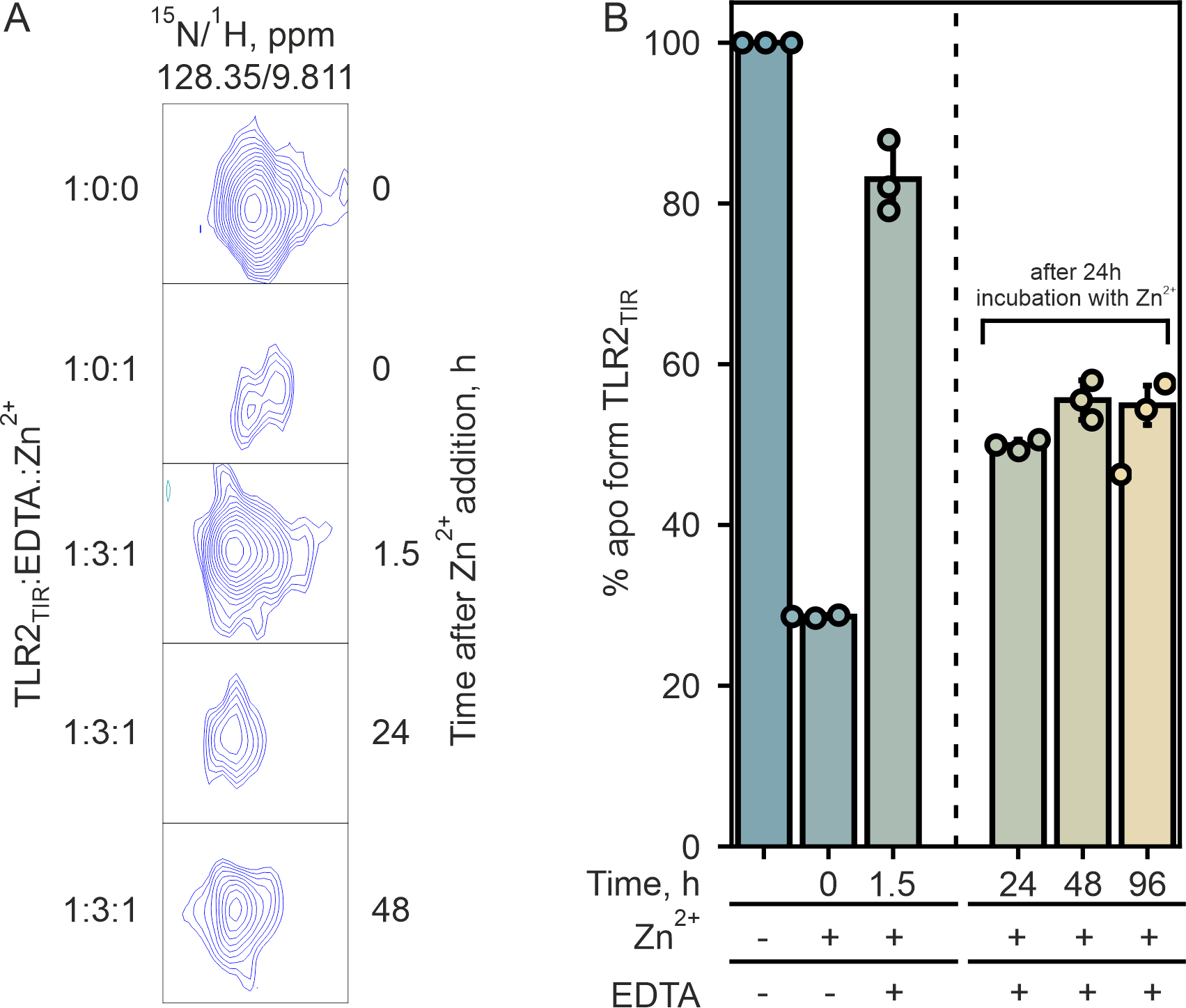


**Fig. S15.** **EDTA competition assay.** **A.** Slices of ^1^H,^15^N-HSQC NMR spectra of the 128.35/9.811 signal. The asterisk (*) indicates that the chelator was added 24 h after incubation of the protein with zinc ions. **B.** NH-Signal intensities of apo TLR2_TIR_ (%) in ^1^H,^15^N-HSQC spectra upon addition of Zn. Error bars indicate the standard deviations.

**
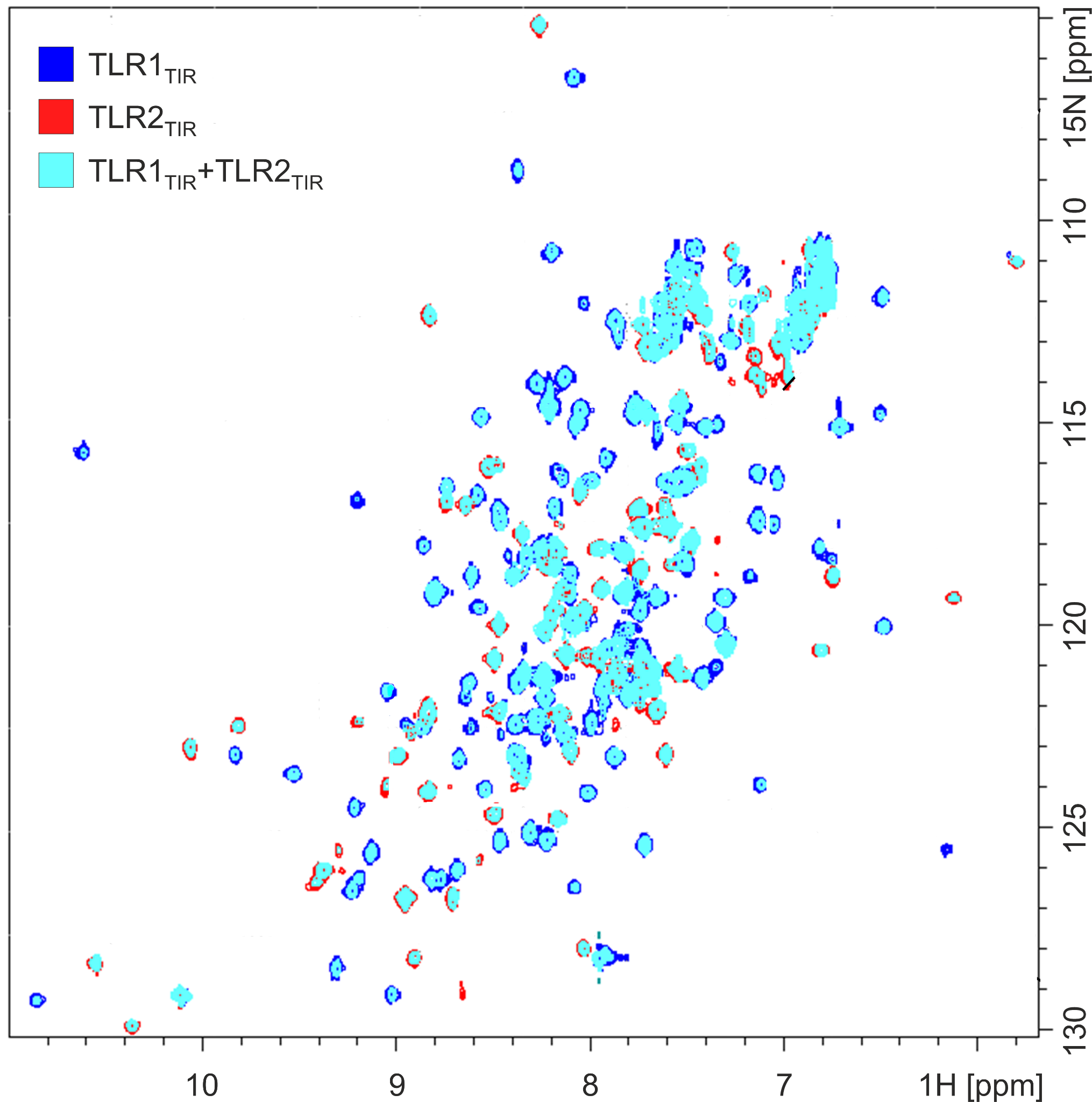
**

**Fig. S16. Analysis of interaction between TLR1_TIR_ and TLR2_TIR_**. Overlay of ^1^H,^15^N-HSQC spectra of TLR1_TIR_ (blue), TLR2_TIR_ (red), and equimolar mix of TLR1_TIR_ and TLR2_TIR_ (cyan).


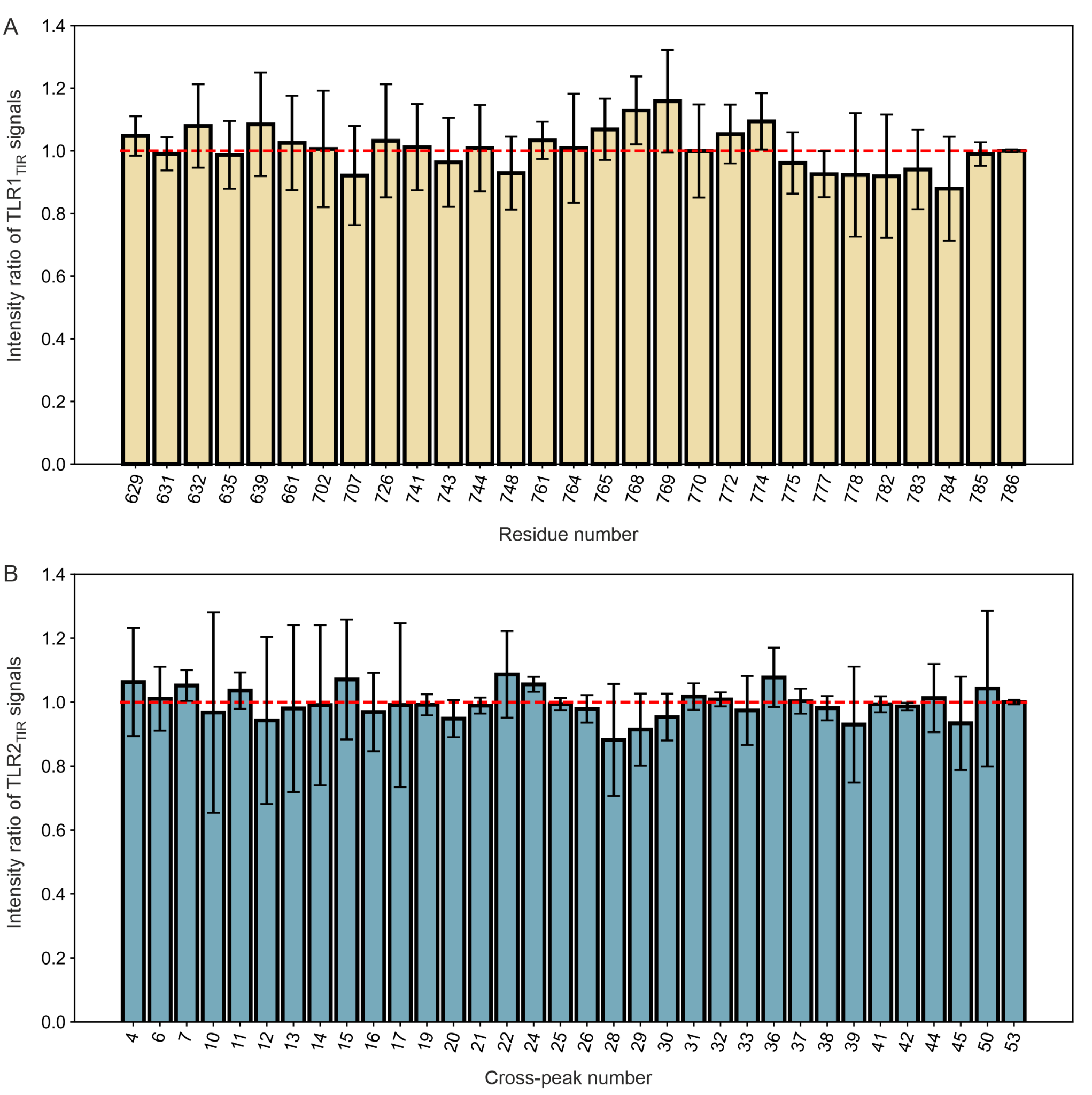


**Fig. S17.** **Comparison of TLR1_TIR_/TLR2_TIR_ signal intensities in ^1^H,^15^N-HSQC spectra before and after protein mixing**. A - The ratio of the signal intensity of TLR1_TIR_ before and after adding an equimolar amount of TLR2_TIR_. **B -** The ratio of the signal intensity of TLR2_TIR_ before and after adding an equimolar amount of TLR1_TIR_. In both panels, the signal intensities normalized to the protein concentration. Signals with a signal-to-noise ratio below 80:1 were excluded from the analysis. The errors are calculated based on the average noise level.


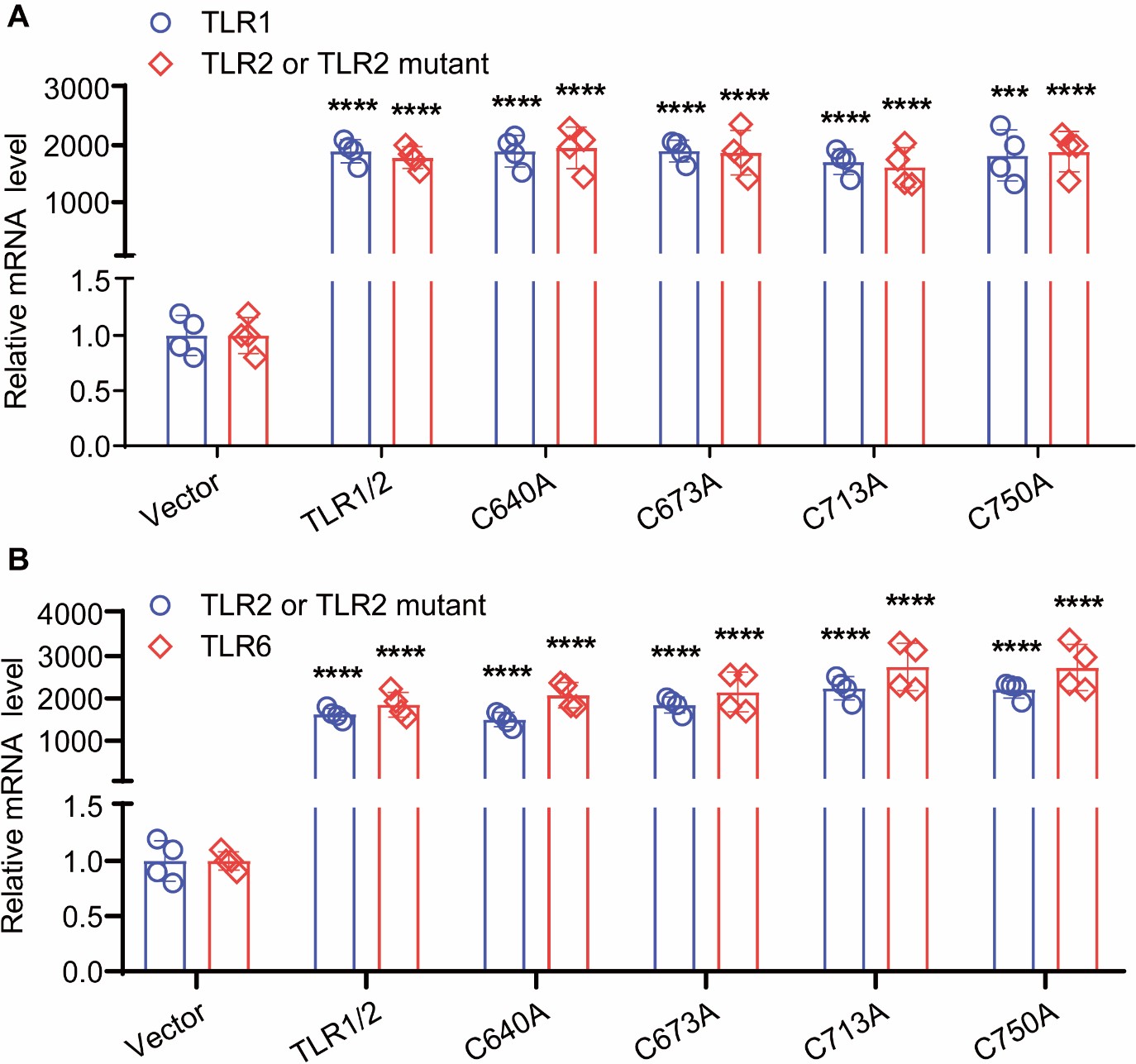


**Fig. S18. Expression levels of TLR2 and 6**. **A** - TLR1 and TLR2 mRNA levels were assessed by qRT-PCR in HEK Blue Null2 cells co-expressing human TLR1 and either wild-type or mutant TLR2. **B** - TLR2 and TLR6 mRNA levels were assessed by qRT-PCR in HEK Blue Null2 cells co-expressing human TLR6 and either wild-type or mutant TLR2. n = 4 independent experiments. Statistical significance is indicated as follows: *** - p < 0.001, and **** - p < 0.0001 with respect to the negative control group. Error bars represent the standard error of the mean.
